# Genomic characterization of novel *Neisseria* species

**DOI:** 10.1038/s41598-019-50203-2

**Published:** 2019-09-24

**Authors:** Kanny Diallo, Jenny MacLennan, Odile B. Harrison, Chisomo Msefula, Samba O. Sow, Doumagoum M. Daugla, Errin Johnson, Caroline Trotter, Calman A. MacLennan, Julian Parkhill, Ray Borrow, Brian M. Greenwood, Martin C. J. Maiden

**Affiliations:** 1 Centre pour les Vaccins en Développement, Bamako, Mali; 2 Department of Zoology, University of Oxford, Oxford, UK; 3 Malawi-Liverpool-Wellcome Trust Clinical Research Programme, College of Medicine, University of Malawi, Blantyre, Malawi; 4 Centre de Support en Santé International, N’Djamena, Chad; 5 Electron Microscopy Facility, Sir William Dunn School of Pathology, University of Oxford, Oxford, UK; 6 Department of Veterinary Medicine, University of Cambridge, Cambridge, UK; 7 Jenner Institute, Nuffield Department of Medicine, University of Oxford, Oxford, UK; 8 Wellcome Trust Sanger Institute, Cambridge, UK; 9 Vaccine Evaluation Unit, Public Health England, Manchester, UK; 10 London School of Hygiene & Tropical Medicine, London, UK

**Keywords:** Microbial ecology, Bacterial genetics, Meningitis

## Abstract

Of the ten human-restricted *Neisseria* species two, *Neisseria meningitidis*, and *Neisseria gonorrhoeae*, cause invasive disease: the other eight are carried asymptomatically in the pharynx, possibly modulating meningococcal and gonococcal infections. Consequently, characterizing their diversity is important for understanding the microbiome in health and disease. Whole genome sequences from 181 *Neisseria* isolates were examined, including those of three well-defined species (*N. meningitidis; N. gonorrhoeae;* and *Neisseria polysaccharea*) and genomes of isolates unassigned to any species (*Nspp)*. Sequence analysis of ribosomal genes, and a set of core (cgMLST) genes were used to infer phylogenetic relationships. Average Nucleotide Identity (ANI) and phenotypic data were used to define species clusters, and morphological and metabolic differences among them. Phylogenetic analyses identified two polyphyletic clusters (*N. polysaccharea* and *Nspp*.), while, cgMLST data grouped *Nspp* isolates into nine clusters and identified at least three *N. polysaccharea* clusters. ANI results classified *Nspp* into seven putative species, and also indicated at least three putative *N. polysaccharea* species. Electron microscopy identified morphological differences among these species. This genomic approach provided a consistent methodology for species characterization using distinct phylogenetic clusters. Seven putative novel *Neisseria* species were identified, confirming the importance of genomic studies in the characterization of the genus *Neisseria*.

## Introduction

The genus *Neisseria* includes a number of species that colonize the epithelium of animals^1^. Human-restricted species are Gram-negative, oxidase and catalase positive bacteria with two different morphologies: coccoid (*Neisseria cinerea, Neisseria gonorrhoeae, Neisseria polysaccharea*, *Neisseria lactamica*, *Neisseria meningitidis*, *Neisseria mucosa*, *Neisseria oralis and Neisseria subflava*); and rods (*Neisseria elongata* and *Neisseria bacilliformis*). All are asymptomatic commensal inhabitants of the mucosa, but two are also potentially pathogenic species: (i) *N. meningitidis*, which colonizes the oropharynx, sometimes causing meningitis and/or septicaemia; and (ii) *N. gonorrhoeae*, which typically inhabits the genitourinary tract, causing local and occasionally systemic gonococcal disease^2^. The remaining eight species are generally harmless inhabitants of the oro- and/or nasopharynx.

Conventional *Neisseria* phenotypic taxonomy reliably identifies the three species most studied at the time of writing: *N. meningitidis*; *N. gonorrhoeae*; and *N. lactamica*. This approach has, however, presented difficulties in defining and identifying other species^3^, which has led to the application of molecular approaches such as DNA-DNA hybridization (DDH)^3^ and phylogenetic analyses of nucleotide sequence data of the 16S ribosomal gene^4^. Analysis with single loci, such as 16S rRNA genes, poorly differentiate *Neisseria* species, due to distortions resulting from low resolution and horizontal genetic transfer (HGT)^5^. Phylogenies based on multi-locus sequence analysis (MLSA)^4^, such as (i) the seven loci used in Multi Locus Sequence Typing (MLST)^5,6^, (ii) the 53 ribosomal genes of ribosomal MLST (rMLST)^7^, and (iii) the 246 loci of the *Neisseria* genus core genome MLST (cgMLST)^3^, have greatly improved *Neisseria* species classification. These approaches led to the reclassification of some *Neisseria* species, with the consolidation of *N. subflava* biovar *sublfava*, *perflava* and *flava*, and *Neisseria flavescens* into a single species named *Neisseria subflava*. Isolates previously classified as *Neisseria sicca* were identified as variants of *N. mucosa*^3^ and “*Neisseria mucosa var. heidelbergensis*”, was shown to be genetically identical to *Neisseria oralis*^8^. A single-locus sequence species identification assay, which uses a fragment of the ribosomal gene *rplF*, can identify different *Neisseria* species reliably, but as this is a single locus test, it is not on its own an appropriate tool for the definition of novel species^9^. Measuring the Average Nucleotide Identity (ANI) between genomes pairs has also facilitated bacterial taxonomic research and been proposed as an alternative to DDH^10,11^, which is highly specialized, labor intensive and difficult to standardize and compare among laboratories^12^.

Non-pathogenic *Neisseria* (NPN) are rarely isolated and characterized, mostly because they seldom cause invasive disease, except in immunocompromised individuals^1^; however, their possible role in modulating nasopharyngeal carriage of *N. meningitidis* has increased interest in them. Several studies have indicated that nasopharyngeal colonization with *N. lactamica* can be protective against *N. meningitidis* infection^13–15^ and that *N. lactamica* has potential to be used in the design an outer-membrane vesicle (OMV) meningococcal vaccines^16^. *Neisseria* are known to be abundant in the human microbiome^17^, reinforcing the importance of understanding the diversity of this genus. This has been exemplified by the findings that: (i) in both high-income countries and in the African meningitis belt, the incidence of carriage of *N. meningitidis* increases with age as that of *N. lactamica* declines^13,18,19^; (ii) the risk factors for carrying *N. meningitidis* in the African Meningitis Belt (AMB) are inversely related to those of carrying NPNs^20^; and (iii) the microbiota has a major influence on other diseases, including pneumonia^21^ and cystic fibrosis^22,23^.

*N. polysaccharea* has been described as polyphyletic, potentially containing more than one species. In particular, isolate 15883, identified by Berger in 1985 in Germany^24^, has been proposed as a new species^3,25^. The increase in the number of non-*N. meningitidis* carriage isolates collected in previously under-sampled and geographically distinct regions^20^, combined with the concomitant decrease in the cost of whole genome sequencing (WGS), have allowed an increasing number of NPN isolates to be sequenced, although numbers remain low compared to *N. meningitidis* and *N. gonorrhoeae*.

Based on the phylogeny of partial *rplF* nucleotide sequences, isolates genetically close to isolate 15883, were identified in MenAfriCar carriage studies, suggesting that this putative novel species, which had not been found since Berger, could be present in the African continent^20^. Similar non-defined isolates were identified in other studies conducted in and outside of the African meningitis belt^25–28^ and in the UKMenCar4 carriage study^29^. Here, phylogenetic relationships of members of the human-restricted *Neisseria* species were examined using WGS data to characterize their genetic and phenotypic diversity and clarify the identity of isolates belonging to the Neisseria genus but currently of unknown species.

## Results

### Hierarchical gene-by-gene analysis identifies polyphyletic groups

**Figure 1:**
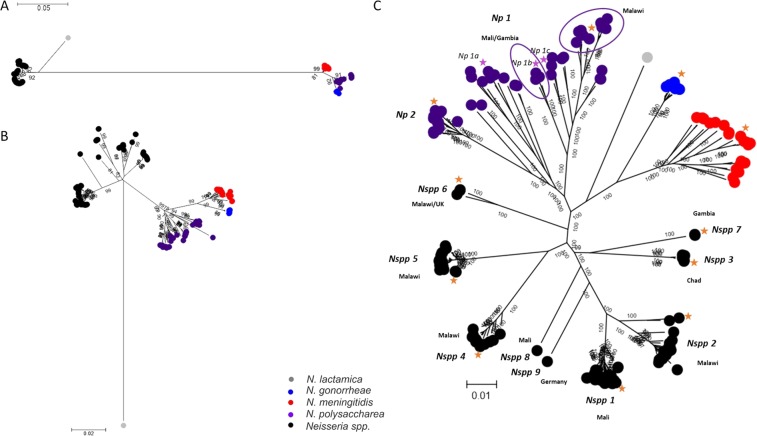
Hierarchical gene by gene analysis. Maximum-likelihood trees generated from the aligned sequences of *f_rplF* (**A**); the concatenated sequences of 51 rMLST loci (**B**) and the concatenated sequences of the 1114 complete cgMLST loci out of the 1441 (**C**). The orange stars correspond to the isolates selected in the first ANI analysis (Table 2) and the purple stars to those added in the ANI analysis done only among the *N. polysaccharea* clusters. The purple circles group *Np* isolates from specific geographical location. Countries of isolation of *Nspp* clusters are indicated. *Np: N. polysaccharea* and *Nspp: Neisseria spp*.

WGS data of the 181 isolates included in this study (Supplemental Table 1) permitted an hierarchical approach to the analysis of bacterial isolate relationships, with increasing numbers of loci providing higher resolution (Fig. 1). A maximum-likelihood phylogeny generated from *f_rplF* sequence data identified four clusters including monophyletic *N. meningitidis* and *N. gonorrhoeae* clusters, and more diverse *N. polysaccharea* and *Nspp* isolates (Fig. 1A). Eighteen *f_rplF* alleles were identified: two among *N. gonorrhoeae* (*f_rplF* alleles 5 and 7); five among *N. meningitidis* (*f_rplF 1*, 2, 3, 4 and 8); six among *N. polysaccharea* (*f_rplF* alleles 9, 39, 44, 63, 90 and 120); and six among *Nspp* isolates (*f_rplF* alleles 16, 62, 69, 70, 79 and 146).

A phylogeny reconstructed from the sequences of 51 ribosomal loci identified distinct clusters among the *Nspp* isolates, each separated by long branches. This also confirmed the polyphyletic relationships of the *N. polysaccharea* isolates (Fig. 1B). A total of 115 rMLST profiles were identified: 9 among *N. gonorrhoeae* isolates; 18 among *N. meningitidis* isolates, with an additional isolate missing two loci and for which an rMLST profile could not be defined; 39 among *N. polysaccharea* isolates; and 49 among *Nspp* isolates. A network analysis using rMLST, which included additional genomes from the remaining human-restricted *Neisseria*^3^, showed that these *Nspp* isolates were distinct from other known species (Supplemental Fig. 1).

Nine distinct clusters were observed in a maximum-likelihood phylogeny derived from the 1114 cgMLST loci with complete sequences for all isolates; the cgMLST was defined for this study as explained in the “genomic diversity” heading of the method section. These were provisionally designated *Nspp* 1 to *Nspp* 9. At least two clusters could be differentiated among the *N. polysaccharea* isolates, designated *N. polysaccharea* 1 and *N. polysaccharea* 2 (Fig. 1C). Phylogeographical clustering was observed in both *Nspp* and *N. polysaccharea* groups as follows: (i) all the isolates from *Nspp* 1 (N = 36) and *Nspp* 8 (N = 1) were collected in Mali; (ii) *Nspp* 2 (N = 20), *Nspp* 4 (N = 14) and *Nspp* 5 (N = 21) were collected in Malawi; (iii) *Nspp* 3 (N = 9) came from Chad; (iv) *Nspp* 7 (N = 2) from The Gambia; and (v) *Nspp* 9 (N = 1) from Germany. The only cluster with isolates from different locations was *Nspp* 6 collected in Malawi (N = 2) and in the UK (N = 1). Similarly, all *N. polysaccharea* 2 isolates were collected in The Gambia (N = 6) and Mali (N = 7), two countries of the African meningitis belt, whereas *N. polysaccharea* 1 included isolates both from Europe and Africa. Even within the *N. polysaccharea* 1 cluster, these remained polyphyletic with clusters separated by deep branches. The ten isolates from Malawi (N = 10) formed a divergent branch and the remaining three African isolates, from Mali (N = 1) and The Gambia (N = 2), clustered more closely together compared to European isolates (Fig. 1C).

### Average nucleotide identity identified seven putative novel Neisseria species

**Figure 2:**
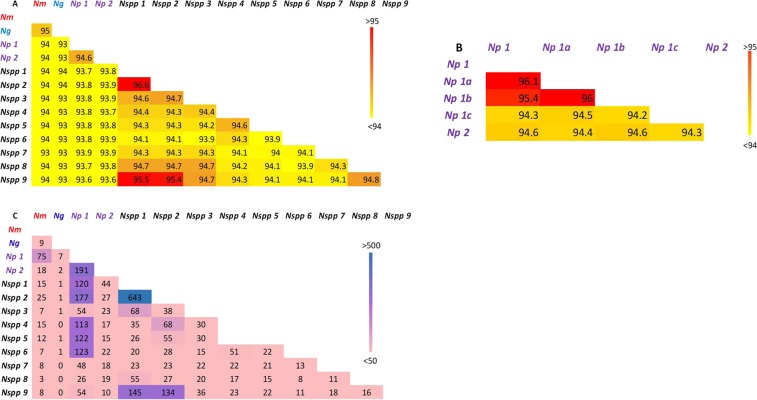
Heatmap of pairwise comparison of the 1114 core genes with complete sequences among the different clusters. Two-way Average Nucleotide Identity using concatenated cgMLST loci sequences among all the clusters (**A**) and only among the *N. polysaccharea* (**B**); ANI measured in percentage are presented with a colour gradient, yellow for values <94 and red for values >95%, which is the threshold above which genomes are considered to be from the species. Pairwise allelic comparison of the cgMLST loci (**C**), Number of loci with at least one allele shared are displayed with a colour gradient, pink for values <50 and purple for values >500. *Nm*: *N. meningitidis*; *Ng*: *N. gonorrhoeae*; *Np*: *N. polysaccharea* and *Nspp*: *Neisseria spp*.

The cgMLST ANI calculated for the 13 representative genomes of the different clusters varied from 93.1% (between *N. gonorrhoeae* and *Nspp* 6 and *N. gonorrhoeae* and *Nspp* 7) to 96.6% (between *Nspp* 1 and *Nspp* 2). Only three pairwise comparisons yielded values >95%, these were between: (i) *Nspp* 1 and *Nspp* 2; (ii) *Nspp* 1 and *Nspp* 9; and (iii) *Nspp* 2 and *Nspp* 9. Using a threshold of 95% ANI, these findings indicate that these should be considered as distinct bacterial species. All the remaining ANI values were lower than 95% (Fig. 2A). Similar results were obtained using whole genome ANI (Supplemental Fig. 2A).

**Figure 3:**
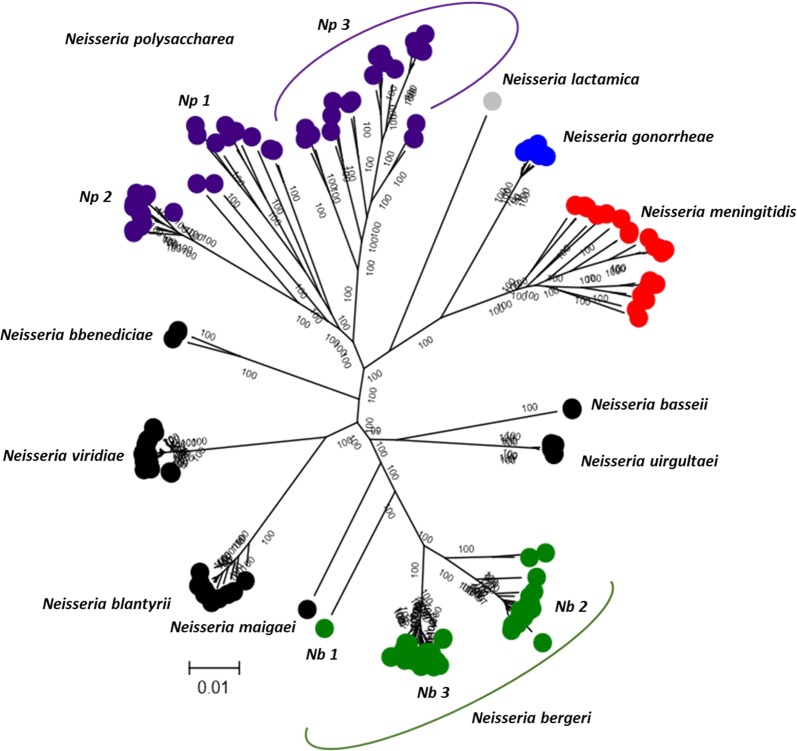
Maximum-likelihood tree of the putative novel *Neisseria* species defined following 95% cgMLST ANI.

Using the 95% threshold, the ANI data were used to classify the *Nspp* isolates into seven distinct species groups, each of which assigned a proposed species name:

1. 57 isolates including clusters *Nspp* 1, *Nspp* 2 and *Nspp* 9; the name *Neisseria bergeri sp. nov*. is proposed for these organisms, after the late Ulrich Berger who isolated the first strain (type strain: 15883; pubMLST ID: 5193) in Germany in 1984^24^.
2. The *Nspp* 8 cluster comprised a single isolate collected in Mali in 2010; the name “*Neisseria maigaei*” is suggested, after the late Aziz Maiga who played an important role in the isolate characterization of the MenAfriCar study in Mali.
3. The name *Neisseria uirgultaei sp. nov*. is proposed for the nine isolates from cluster *Nspp* 3 (type strain: 12_13955_XS2_1; pubMLST ID: 43129), after Sir Brian Greenwood, the director of the MenAfriCar project (greenwood is *uirgulta* in Latin).
4. As only two isolates have been identified in *Nspp* 7, the name “*Neisseria basseii*” is suggested, after the town of Basse in The Gambia, where the isolates were collected.
5. The name *Neisseria blantyrii* is proposed for the 14 *Nspp* 4 isolates (type strain: M-041; pubMLST ID: 46402), after the city of Blantyre in Malawi where the isolates were collected.
6. *Neiseria viridiae sp. nov*. is proposed for the 21 *Nspp 5* isolates (type strain: M-226; pubMLST ID: 46439), after Jenny MacLennan (*nee* Green) who collected and characterized all the isolates from Malawi and The Gambia included in this study (green is *viridi* in Latin).
7. Finally, the name “*Neisseria benedictiae*” is suggested for the three *Nspp* 6 isolates, after Julia Bennett (bennet is *benedictus* in Latin) who pioneered the genomic work of *N. bergeri* isolates and the speciation of *Neisseria* with cgMLST approaches (Fig. 3).

The pairwise allelic comparison of the 1114 core loci with complete sequences between each defined cluster identified that, despite sharing the same loci, most clusters had distinct alleles, with only small numbers of shared allele between clusters. *Nspp* 1 and *Nspp* 2, however, had the largest number of loci (N = 693), sharing at least one allele, and similarly for *Nspp* 1 and *Nspp* 9 (N = 145) and *Nspp* 2 and *Nspp* 9 (N = 134) (Fig. 2C), consistent with the ANI analysis suggesting their grouping into a single species.

### Neisseria polysaccharea is polyphyletic

The two *N. polysaccharea* clusters, designated *N. polysaccharea* 1 and *N. polysaccharea 2*, had an ANI value of 94.6%, confirming that they can be considered as two distinct species (Fig. 2A). The pairwise allelic comparison based on the two clusters found that *N. polysaccharea* 1 also had large number of loci sharing at least one allele with various clusters (*N. polysaccharea* 2*; Nspp* 1*, Nspp* 2, *Nspp* 4*, Nspp* 5 and *Nspp* 6), suggesting that the cluster was not homogeneous and probably included more than one species. Three additional genomes of the *N. polysaccharea 1* cluster were selected to attempt to characterize this further (Fig. 1C). 95% cgMLST and whole genome ANI (Fig. 2B and Supplemental Fig. 2B) comparison among the genomes of these 5 isolates identified another distinct cluster, *N. polysaccharea 3* (Fig. 3).

### Phenotypic analysis of clusters

Macroscopic analysis of bacterial colony morphology established that colonies from all isolates tested were grey, exhibited γ-haemolysis, and formed a round shape on blood agar (BAP). The putative novel species exhibited edges with a regular shape and smooth texture; however, *Neisseria blantyrii* (*Nspp* 4) visually appeared to be moister. The size range of the colonies varied from 0.1 mm to 1 mm; *N. meningitidis* colony size range extended to 2 mm (Table 1).

**Table 1:** Morphological characteristics of *Neisseria* species colonies, including those of the 7 new species.

Morphology evaluated for colony of each species	Haemolysis	Colour	Texture	Size	Shape	Shape of the edges
*Neisseria bergeri (Nspp9)*	gama	grey	smooth	0.1–1 mm	round	regular
*Neisseria bergeri (Nspp1)*	gama	grey	smooth	0.5–1 mm	round	regular
*Neisseria bergeri (Nspp2)*	gama	grey	smooth	0.5–1 mm	round	regular
*Neisseria maigaei* (*Nspp8)*	gama	grey	smooth	0.2–0.5 mm	round	regular
*Neisseria uirgultaei* (*Nspp3)*	gama	grey	smooth	0.5–1 mm	round	regular
*Neisseria basseii* (*Nspp7)*	gama	grey	smooth	0.5 mm	round	regular
*Neisseria blantyrii (Nspp4)*	gama	grey	smooth/moist	0.2–0.5 mm	round	regular
*Neisseria viridiae (Nspp5)*	gama	grey	smooth	0.5–1 mm	round	regular
*Neisseria benedictiae (Nspp6)*	gama	grey	smooth	0.5–1 mm	round	regular
*Neisseria meningitidis*	gama	grey	moist	0.5–2 mm	round	irregular/merging
*Neisseria polysaccharea*	gama	grey	moist	0.2 mm	round	regular
*Neisseria lactamica*	gama	grey	smooth	1 mm	round	regular

**Figure 4:**
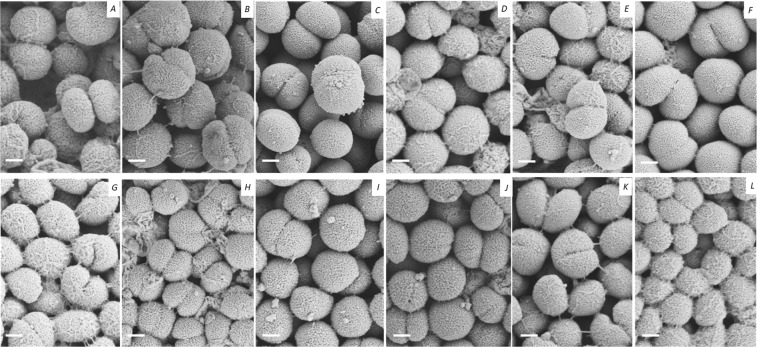
Scanning Electron Microscopy images of the different clusters identified by genomic analysis. A-N. meningitidis, B-N. polysaccharea cluster 2, C-N. polysaccharea cluster 3, D-Nspp 1 (*N. bergeri*), E-Nspp 2 (*N. bergeri*), F-Nspp 3 (*N. uirgultaei*), G-Nspp 4 (*N. blantyrii*), H-Nspp 5 (*N. viridiae*), I-Nspp 6 (*N. benedictiae*), J-Nspp 7 (*N. bassei*), K- Nspp 8 (*N. maigaei*) and L-Nspp 9 (*N. bergeri*). The scale bar represents 0.25 µm.

Scanning electron microscopy (SEM) of representative isolates from each proposed species revealed that they were all coccoid bacteria, with a mix of completely formed diplococci and incompletely formed diplococci, which were characterized by a “heart-shaped” morphology (Fig. 4). Variation over the extracellular structures observed around the cell membrane was identified: for example, isolates from the three sub-clusters of *N. bergeri* (*Nspp* 1, 2, and 9) exhibited filaments covering their extracellular membranes, “pili-like” structures could also be seen connecting cells of “*N. maigaei”* (*Nspp* 8), “*N. blantyrii*“(*Nspp* 4) cells were covered in an intense filamentous network, “*N. viridiae”* (*Nspp* 5) and “*N. benedictiae”* (*Nspp* 6) exhibited visible “bulbs” on the surface of their extracellular membranes. Conversely, “*Neisseria uirgultaei”* (*Nspp* 3) possessed a smooth surface with no visible extracellular elements and “*Neisseria basseii”* (*Nspp* 7) showed few visible filaments and bulbs (Fig. 4).

Biochemical data obtained using Diatabs^TM^ system identified the putative novel species as GGA, ONPG, and Tributyrin negative, Leucine and Proline Aminopeptidase positive. They were also positive for the maltose degradation and negative for lactose degradation. Results were inconsistent for glucose and sucrose when repeated: *N. bergeri* subcluster 1 (*Nb1*; *Nspp* 9) and 2 (*Nb2*; *Nspp* 2), “*N. uirgultaei”* (*Nspp* 3), “*N. basseii*” (*Nspp* 7) and “*N. viridiae”* (*Nspp* 5) being mostly positive for glucose after at least 3 repeats, and *N. bergeri* subcluster 3 (*Nb3*; *Nspp* 1), “*N. maigaei*” (*Nspp* 8)*, “N. blantyrii”* (*Nspp* 4) and “*N. benedictiae”* (*Nspp* 6) mostly negative. Similarly, “*N. viridiae”* (*Nspp* 5) and “*N. benedictiae*” (*Nspp* 6) were mostly positive for sucrose while all the other species were negative. GGA, proline aminopeptidase and lactose/ONPG tests were useful for distinguishing new species from *N. meningitidis* and *N. lactamica*; however, none of these allowed a distinction from *N. polysaccharea* (Table 2). Results obtained from the API®-NH tests showed that all the isolates were positive for the degradation of glucose, fructose, maltose, and saccharose, which contradicted the pattern observed for glucose with the Diatabs^TM^ for some isolates. Similarly, to the Diatabs^TM^ assay, the β-galactosidase, proline aminopeptidase and gamma-glutamyl transferase (GGT) reactions were useful to distinguish putative novel species from *N. lactamica* and *N. meningitidis* but no biochemical test could distinguish them from *N. polysaccharea* (Table 3).

**Table 2:** Biochemical characteristics of putative novel *Neisseria* specie*s* including sugar metabolism and other biochemical properties tested using the Diatabs system.

Diatabs	Glucose	Maltose	Sucrose	Lactose	GGA	LA	PA	ONPG	Tributyrin
*Neisseria bergeri (Nspp9)*	+*	+	−	−	−	+	+	−	−
*Neisseria bergeri (Nspp1)*	−*	+	−	−	−	+	+	−	−
*Neisseria bergeri (Nspp2)*	+	+	−	−	−	+	+	−	−
*Neisseria maigaei* (*Nspp8)*	−	+	−	−	−	+	+	−	−
*Neisseria uirgultaei* (*Nspp3)*	+	+	−	−	−	+	+	−	−
*Neisseria basseii* (*Nspp7)*	+*	+	−	−	−−	+	+	−	−
*Neisseria blantyrii (Nspp4)*	−*	+	−	−	−	+	+	−	−
*Neisseria viridiae (Nspp5)*	+	+	+*	−	−	+	+	−	−
*Neisseria benedictiae (Nspp6)*	−	+	+*	−	−	+	+	−	−
*Neisseria meningitidis*	+	+	−	−	+	+	−	−	−
*Neisseria polysaccharea*	+*	+	−	−	−	+	+	−	−
*Neisseria lactamica*	+	+	−	+	−	+	+	+	−

GGA: Gama-Glutamyl Aminopeptidase; LA: Leucine Aminopeptidase; PA: Proline Aminopeptidase; ONPG: Ortho-Nitrophenyl-β-galactoside; *Nspp*: *Neisseria spp*.

*Variable results upon multiple repeats, the most common result is presented.

**Table 3:** Biochemical characteristics of putative novel *Neisseria* specie*s* including sugar metabolism and additional biochemical properties tested on the API-NH strip.

API-NH	PEN	GLU	FRU	MAL	SAC	ODC	URE	LIP	PAL	βGAL	ProA	GGT	IND
*Neisseria bergeri (Nspp9)*	−	+	+	+	+	−	−	−	−	−	+	−	−
*Neisseria bergeri (Nspp1)*	−	+	+	+	+	−	−	−	−	−	+	−	−
*Neisseria bergeri (Nspp2)*	−	+	+	+	+	−	−	−	−	−	+	−	−
*Neisseria maigaei* (*Nspp8)*	−	+	+	+	+	−	−	−	−	−	+	−	−
*Neisseria uirgultaei* (*Nspp3)*	−	+	+	+	+	−	−	−	−	−	+	−	−
*Neisseria basseii* (*Nspp7)*	−	+	+	+	+	−	−	−	−	−	+	−	−
*Neisseria blantyrii (Nspp4)*	−	+	+	+	+	−	−	−	−	−	+	−	−
*Neisseria viridiae (Nspp5)*	−	+	+	+	+	−	−	−	−	−	+	−	−
*Neisseria benedictiae (Nspp6)*	−	+	+	+	+	−	−	−	−	−	+	−	−
*Neisseria meningitidis*	−	+	+	+	+	−	−	−	−	−	−	+	−
*Neisseria polysaccharea*	−	+	+	+	+	−	−	−	−	−	+	−	−
*Neisseria lactamica*	−	+	+	+	+	−	−	−	−	+	+	−	−

PEN: Penicilinase; GLU: glucose; FRU: fructose; MAL: maltose; SAC: saccharose; URE: urea; LIP: Lipase; PAL: Alkaline Phosphatase; βGAL: β-Galactosidase; ProA: Proline Arylamidase; GGT: Gamma-Glutamyl Transferase; IND: Indole; *Nspp*: *Neisseria spp*.

## Discussion

The concept and definition of species in prokaryotes continues to be subject to debate, as do the best methods for microbial taxonomy^30,31^. Many bacterial species have been defined solely on phenotypic characteristics^4^. For example, *N. meningitidis* and *N. gonorrhoeae*, were defined by their very different clinical manifestations, although they are very closely related and do not meet the 70% DDH criterion necessary to separate them into distinct species taxonomically^32^. The integration of ecological and genetic data has greatly improved classification. With the advent of high-throughput nucleotide sequencing studies, multi locus sequence analysis (MLSA) became an approach of choice^33^. The analysis of the seven MLST loci has proven to be sufficient to distinguish *N. meningitidis, N. gonorrhoeae*, and *N. lactamica*^34^, for example, which single-locus 16S RNA sequence analysis has failed to achieve^3^. Although housekeeping genes have predominantly been used, there have been no specific guidelines on the loci or the number of loci to be chosen for MLSA. The increased availability of whole or nearly complete genome sequence data allows species characterization at a higher resolution, as demonstrated through the use of the *Neisseria* genus cgMLST to identify multiple *Neisseria* species^3^.

The phylogenomic approach^35^ adopted here combines rMLST, MLSA, and ANI scores, using nucleotide sequence data of core genes, supplemented with a quantitative measure of genetic relatedness (Fig. 2A,B). This allows phylogenetic relationships to be inferred among the collection of isolates under study (Fig. 1C). The 95% cgMLST ANI cut-off identified seven distinct species (Fig. 3); however, three clusters identified by phylogenomic analyses were indistinguishable by ANI and were therefore grouped into a single species named *N. bergeri sp. nov*. These three clusters can be described as subspecies (*Nb* Subspecies 1, 2, and 3). Three putative novel *Neisseria* species were assigned names, as clusters contained more than three representative isolates: *N. uirgultaei sp. nov., N. blantyrii sp. nov*., and *N. viridiae sp. nov*. The remaining clusters had fewer than three isolates and therefore will not be formally named; however, the species names (“*N. maigaei*”, “*N. basseii*” and “*N. benedictiae*”) are proposed for future reference. Identical ANI results were obtained using the full draft WGS (Supplemental Fig. 2A), confirming that using genes core to these human-restricted *Neisseria* species provided optimal genomic resolution with exclusion of intergenic regions and accessory genes not affecting results. Congruence between ANI results and cgMLST allelic comparison (Fig. 2A,C), suggests that allelic diversity plays an important role in the speciation process, alongside gene presence/absence. The low number of shared alleles confirms the infrequency of cross-species recombination as previously observed in a study comparing *N. meningitidis, N. lactamica*, and *N. gonorrhoeae*^36^. Analyses identified distinct clusters among the *N. polysaccharea* isolates included in this study: two phylogenetic clusters, *N. polysaccharea* 2 and *N. polysaccharea* 3, could be reclassified as distinct species based on the 95% cgMLST and ANI (Fig. 2B and Supplemental Fig. 2B). However, a subset (*N. polysaccharea* 1) remained polyphyletic.

A major limitation when undertaking taxonomic studies is ensuring the natural diversity of the bacterial population has been appropriately sampled. Studies are often geographically limited, leading to an underestimation of diversity. The different nasopharyngeal carriage studies undertaken in the UK^29,37^, countries of the African Meningitis Belt^19,26,38^, and in Malawi (outside the belt)^28^ have led to the collection of a large number of *Neisseria* isolates; however, the NPNs were not always characterized at the genomic level. Our results indicate a phylogeographic clustering of genotypes, with isolates from the same country clustering together (Fig. 1C). Even though most recent carriage studies, done in the UK and in African countries, used the same laboratory methodology, most of the previously non-speciated isolates were recovered in Africa with only one *Nspp* found in the UK, suggesting a potentially greater diversity of *Neisseria* species in African countries than in the UK. However, differences in the age groups targeted, the whole population (AMB and Malawi) as opposed to 15–19 years old (UK), may have played a role in the differences observed.

WGS analysis of additional carried *Neisseria* isolates from various geographical regions will improve the taxonomy of this genus. Isolates previously characterized as “*N. bergeri*”, based on nucleotide sequences of the *rplF* locus^20^, included additional putative novel *Neisseria* species, such as those identified in this study. Genome sequences obtained from the Malian collection of the MenAfriCar project found that most of *Nspp* genomes were classified as *N. bergeri sp. nov*. using the 95% cgMLST ANI analysis presented here; however, one was found to form a distinct putative species, “*N. maigaei”*. Although the sequencing of the *f_rplF* locus provides species identity for previously defined species, our study confirms that it does not provide sufficient resolution for the characterization of new species. Each of the clusters identified had distinct *f_rplF* alleles except *N. benedictiae* (*Nspp* 6) and *N. basseii* (*Nspp* 7) which shared allele 69, consistent with recent HGT or shared ancestry.

All the putative novel species and the *N. polysaccharea* isolates were indistinguishable using standard biochemical tests, despite being clearly distinct by 95% cgMLST ANI. However, additional tests including biochemical analyses, pH, or chemotaxonomy (analysis of chemical composition of the cell) combined with a larger numbers of isolates, including additional type strains, may identify further differences^39^. Phenotypic tests are labor intensive, and the interpretation of the results can be subjective. The Diatabs^TM^ tests were, overall, more straightforward than API®-NH tests, which have a strict McFarland Standard concentration. Sugar degradation by the Diatabs^TM^ were accurate for the *N. meningitidis* and *N. lactamica* controls but were elsewhere unstable with repeats giving different results. On the other hand, the degradation of sugars assessed by the API®-NH were highly reproducible, but failed to give the expected results for *N. meningitidis*^24^. Coccoid cells, some of which exhibiting distinct extracellular membrane structures, were visible following SEM analyses (Fig. 4). After 20 hours of incubation, most of the putative novel species had “Heart-shaped” cells (Fig. 4). These cells were still visible after 48 h of incubation (data no shown) but additional time points may establish whether these forms were intermediate shapes of coccoid cells or remained distinctive. Overall, phenotypic tests were inconclusive.

The genomic analyses presented here, allowed putative novel species predominantly found in African countries to be identified. Determining the broader implications of these findings will require more sampling, genomic characterization and analyses of the interactions of these different species in the nasopharynx. This information will determine any role that they play in modulating carriage and invasion by *N. meningitidis*. Future microbiome and immunological studies will be crucial to shed further light on the dynamics of carriage, but genomic characterizations of the isolates will be necessary to adequately assess the diversity. Another novel, non-human-restricted, *Neisseria* species has been recently identified in the US, indicating the potential for other unsampled species^40^.

Results from this study suggest that *Neisseria* diversity may be greater in countries outside the AMB, for example Malawi, than within the belt. This could have important implications regarding the impact of those species in modulating the carriage and invasiveness of *N. meningitidis*, as the disease epidemiology is known to be very different between these two regions of Africa; however, it is likely that analysis of more genome sequences of the *Neisseria* isolates from the AMB, such as those from the other countries sampled alongside Mali and Chad during the MenAfriCar project^20^, will improve our understanding of the diversity seen within the AMB and allow more adequate hypotheses to be formulated. The analytical methods developed here provide a framework for future speciation endeavors and can easily be repeated to incorporate additional genomes when available. It is now evident that the diversity of human restricted *Neisseria* had been underestimated and more carriage studies using genomic methods should be encouraged to define a more accurate picture of the genus.

## Materials and Methods

### Isolates and genomes

#### Isolate collection

A total of 107 *Neisseria* isolates unclassified to species level (*Nspp*) were identified in the pubMLST *Neisseria* database; some of these had been recorded as “*Neisseria bergeri”*, following preliminary analysis^20,25,28^. The majority of the *Nspp* were collected in carriage studies performed in Africa and included: (i) 57 isolates collected from Ndirande, Blantyre, Malawi, from^27,28,41^; (ii) 37 isolates collected in Mali and 9 from Chad^20,38^; and (iii) 2 isolates obtained in Basse, Gambia^26^. Two additional isolates were collected in Europe: the first in Germany in^24^; and the second in the United Kingdom^29^.

#### DNA preparation, Genome sequencing and assembly

All the African *Nspp* isolates were sequenced *de novo* using the Illumina MiSeq platform. Isolates were cultured on blood agar plates (BAP) and incubated overnight for 16–24 hours at 37 °C, in 5% CO_2_. For 96 of the isolates, genomic DNA was extracted using the Wizard genomic DNA Purification kit (Promega) following the manufacturer’s instructions; for the remaining nine isolates from Chad, DNA was extracted using the DNeasy Blood and Tissue kit (Qiagen), as previously described^42^. DNA samples were sequenced by pair-end sequencing on an Illumina HiSeq, with read lengths of 100 (N = 55), 125 (N = 46) and 150 (N = 4) base pairs at the Wellcome Trust Sanger Institute, using previously described methods^43^.

Resultant short reads were assembled using the Velvet genome assembly program (v1.2.08)^44^: all odd-numbered kmer lengths between 21–99, 21–124 and 21–149 were sampled for read lengths of 100, 125, and 150 base pairs respectively, using the VelvetOptimiser software (v2.2.4)^45^ to automatically establish the optimal assembly parameters for Velvet (default optimization functions used). The assembled contigs were deposited in the *Neisseria* PubMLST database^46^ which uses the Bacterial Isolate Genome Sequence Database (BIGSdb) software^47^ and to the European Nucleotide Archive (Accession numbers in Table 1). Draft genomes were automatically curated at all loci defined in the database using the BLAST algorithm^48^ and a sequence similarity threshold of >98%, allowing rapid annotation of known alleles and sequences which were very similar to defined loci^43^. Manual verification was then performed for variable loci, such as those containing internal stop codons, frame shifts or those with sequence similarities lower than 98%. Incomplete gene sequences at the beginning or end of a contig were identified as such.

#### Additional genomes

In addition to the 105 African *Nspp* sequenced *de novo*, and the two European strains previously sequenced and deposited into the http://pubMLST.org/neisseria database, 74 other genomes deposited in the database were chosen as described below, to complete the genome collection analyzed in this study. These represented three known human-associated *Neisseria* species and included all the genomes available at the time of analysis for *N. polysaccharea* (N = 45), all but one finished genomes of *N. gonorrhoeae* (N = 9) and representative genomes of the most common clonal complexes of *N. meningitidis* (N = 20)^43^, with a preference for finished genomes when available. Additional information and meta-data for each of the 181 genomes are available in Supplemental Table 1.

### Genomic diversity

#### Phylogenetic analysis

A hierarchical gene-by-gene approach to Whole Genome Sequence (WGS) analysis was applied to all 181 genomes, including the analysis of: (i) *f_rplf*, a fragment of the ribosomal gene *rplF*^9^, (ii) 51 of the 53 rMLST loci (BACT0060 (*rpmE*) and BACT0065 (*rpmI*) were excluded as they are known to be paralogous in *Neisseria* species); and (iii) a newly defined cgMLST scheme (Human-restricted *Neisseria* cgMLST v1.0), including 1441 loci present in at least 95% of isolates in this collection. All the sequences were aligned using MAFFT in pubMLST^46,49^.

Two methods where used in parallel to generate the list of core loci using: (i) the Genome Comparator tool of PubMLST was used to compare all genomes, using the *N. meningitidis* finished genome FAM18 (Accession number: AM421808) as a reference with parameters set at a minimum identity of 70%, a minimum alignment length of 50%, a BLASTN word size of 20 base pair and a core threshold of 95% (paralogous loci were excluded from this analysis and variable loci aligned and concatenated into a single sequence for each genome); (ii) Proteinortho, assembled draft genomes were downloaded from PubMLST for each isolate and used as input files for the annotation program Prokka, using default settings with the option “–addgenes” and “–compliant”^50^. The protein sequence outputs of Prokka were used as input files for Proteinortho, with default settings, to detect orthologous group of genes^51^.

Both methods generated a list of core loci present in 95% of the genomes included in the analysis; 1421 loci were identified by Genome comparator and 1366 by Proteinortho. An inclusive list of 1441 loci including any that were considered core to 95% of isolates by both methods was generated and used in the phylogenetic analysis. Within these 1441 loci, 1114 had complete sequences in all the genomes, whereas the remaining 327 fell at the beginning or the end of a contig in at least one genome and were classified as incomplete genes and excluded from the phylogenetic analysis (Supplemental Table 2).

Phylogenetic analyses were performed for *f_rplF*, rMLST, and cgMSLT loci using a finished genome of *Neisseria lactamica* (pubMLST ID: 8851/isolate ID: 020-06) as an out-group. The *f_rplF* and rMLST phylogenies were generated in MEGA6 using the Maximum likelihood method with the following options: General Time Reversible model^52^, 100 bootstrap replications, Gamma distributed rate with Invariant sites and 5 discrete Gamma Categories. The cgMLST phylogeny was generated in FastTree^53^ using the General Time Reversible model (“-gtr”) and all other defaults options. Tree annotations were performed in MEGA6^54^.

Another rMLST phylogeny was reconstructed with the Neighbor Joining model in MEGA6 using the 181 genomes previously described and all 40 additional human-restricted *Neisseria* species included in the paper published by Bennett *et al*., which included the available type strains genomes^3^.

Pairwise allelic variations at the core genes, between each identified clusters, were assessed using the allele-overlap script comparing alleles of each gene between two defined sets of genomes^55^.

### Statistical methods

#### Average nucleotide identity

The Average Nucleotide Identity (ANI) was calculated by comparing concatenated sequences of cgMLST loci from representative isolates of each phylogenetic cluster of interest: 13 isolates for the analysis of the whole phylogeny and 5 for the further characterization of the *N. polysaccharea* cluster. An online ANI tool, “ the ANI calculator”^56,57^ was used to calculate a two-way ANI between each pair of selected isolates using the method described previously^11^. Pairs of isolates with ANI values higher than 95% were considered as indistinguishable at the species level as suggested by the program. The analysis was also computed using the whole genomes sequences of the isolates.

### Phenotypic analyses

Phenotypic characteristics of the clusters identified were evaluated using representative isolates from cluster *Nspp1-9*, *Np1-2*, and three positive controls: (i) *N. meningitidis* (pubMLST ID: 35956/isolate ID: Z1318)^58^; (ii) *N. polysaccharea* (pubMLST ID: 19095/isolate ID: CCUG 18031)^59^; and (iii) *N. lactamica* (pubMLST ID: 8851/isolate ID: 020-06)^59^ (Supplemental Table 1). Each isolate was cultured on Columbia Horse Blood Agar plates (Oxoid) and incubated at 37 °C for 16 to 24 hours, in an atmosphere containing 5% CO_2_. A single isolated colony was then sub-cultured under the same conditions for 20 hours before carrying out the tests described below.

#### Morphological analysis

Macroscopic assessment. Cell morphology was characterized, and the color, texture, shape and colony contour were visually assessed after 20 h of incubation. The size of individual colonies was measured using a graduated ruler.

Scanning Electron microscopy (SEM). Sub-cultured isolates were prepared for SEM after 20 hours of incubation, following adaptation of a protocol previously described^60^. Briefly, a piece of agar with bacterial cells attached was excised and fixed with a 2.5% glutaraldehyde solution in 0.1 M Phosphate buffer and incubated at room temperature for 1 hour. Specimens were then rinsed in 0.1 M Phosphate buffer and post-fixed with 1% osmium tetroxide for 1 hour. Specimens were then rinsed in Mili-Q water and dehydrated using an ethanol series: 50% for 10 min, 70% overnight (stored at 4 °C), 90% for 10 min, 95% for 10 min, 100% for 20 min and in pure ethanol three times for 20 min each. Ethanol was removed, and the samples were dried by incubating specimens in a 50% Hexamethyldisilazane (HMDS) and 50% ethanol solution for 15 min, followed by incubation in pure HMDS for an additional 15 min. Once HMDS was removed, samples were left in a fume hood overnight to allow HMDS fumes to evaporate. Samples were mounted on SEM stubs with a conductive carbon adhesive, then coated with 10–15 nm of gold using a Quorum Technologies Q150R ES sputter coater and were imaged with a Zeiss Sigma 300 field emission gun SEM at an accelerating voltage of 2 kV.

#### Sugar degradation and other biochemical tests

Oxidase tests were conducted with the oxidase test disks (Sigma-Aldrich) following the manufacturer’s instructions. Catalase tests were undertaken by putting the bacteria in contact with a drop of hydrogen peroxide; the formation of bubbles was considered a positive reaction. Haemolysis was assessed for each sample by visual inspection of the cells on BAP.

Diatabs^TM^ (Rosco Diagnostica) were used according to the manufacturer’s instructions to test for sugar degradation of glucose, maltose, sucrose, and lactose, and other selected microbial enzymatic properties including: the ability to enzymatically hydrolyse aminopeptidase substrates like gamma glutamyl aminopeptidase (GGA), leucine aminopeptidase and proline aminopeptidase; the hydrolysis activity of the enzyme β-galactosidase on the orthonitrophényl-β-galactoside (ONPG) substrate; and the enzymatic hydrolysis of tributyrin into butyric acid and glycerol often used for the differentiation of *Moraxella catarrhalis* from *Neisseria* species. The results were assessed following the instructions of the test (Supplemental Table 3).

The API-NH® kit (Biomerieux) for *Neisseria* characterisation was also tested in parallel, following the manufacturer’s instructions (Supplemental Table 4).

### Accession number(s)

The Accession numbers for the *de novo* sequenced isolates are provided in Table 1. Accession numbers or reference are provided for the other genomes. All genomes will be made available on the pubMSLT database upon publication of the manuscript.

## Supplementary information

Supplemental figures and tables
