# Supplementary material for: Genomic characterization of novel Neisseria species
Source: Sci Rep. 2019 Sep 24;9:13742. doi: 10.1038/s41598-019-50203-2 (PMC6760525; doi:10.1038/s41598-019-50203-2)
Supplement: Supplementary file 1 — Supplemental figures and tables [file 41598_2019_50203_MOESM1_ESM.docx]

Title: Genomic characterization of novel *Neisseria* species

Running title: *Neisseria* species genomic characterization

Authors: Kanny Diallo,^a,b^^#^ Jenny MacLennan,^b^ Odile B Harrison,^b^ Chisomo Msefula,^c^ Samba O Sow,^a^ Doumagoum M Daugla,^d^ Errin Johnson,^e^ Caroline Trotter,^f^ Calman A MacLennan,^g^ Julian Parkhill,^h^ Ray Borrow,^i^ Brian M Greenwood,^j^ and Martin CJ Maiden^b^

^a^Centre pour les Vaccins en Développement, Bamako, Mali; ^b^Department of Zoology, University of Oxford, Oxford, UK ; ^c^Malawi-Liverpool-Wellcome Trust Clinical Research Programme, College of Medicine, University of Malawi, Blantyre, Malawi;^d^Centre de Support en Santé International, N’Djamena, Chad; ^e^ Electron Microscopy Facility, Sir William Dunn School of Pathology, University of Oxford, Oxford, UK; ^f^ Department of Veterinary Medicine, University of Cambridge, Cambridge, UK; ^g^Jenner Institute, Nuffield Department of Medicine, University of Oxford, Oxford, UK ; ^h^Wellcome Trust Sanger Institute, Cambridge, UK; ^i^Vaccine Evaluation Unit, Public Health England, Manchester, UK; ^j^London School of Hygiene & Tropical Medicine, London, UK.

**Supplemental Figures**


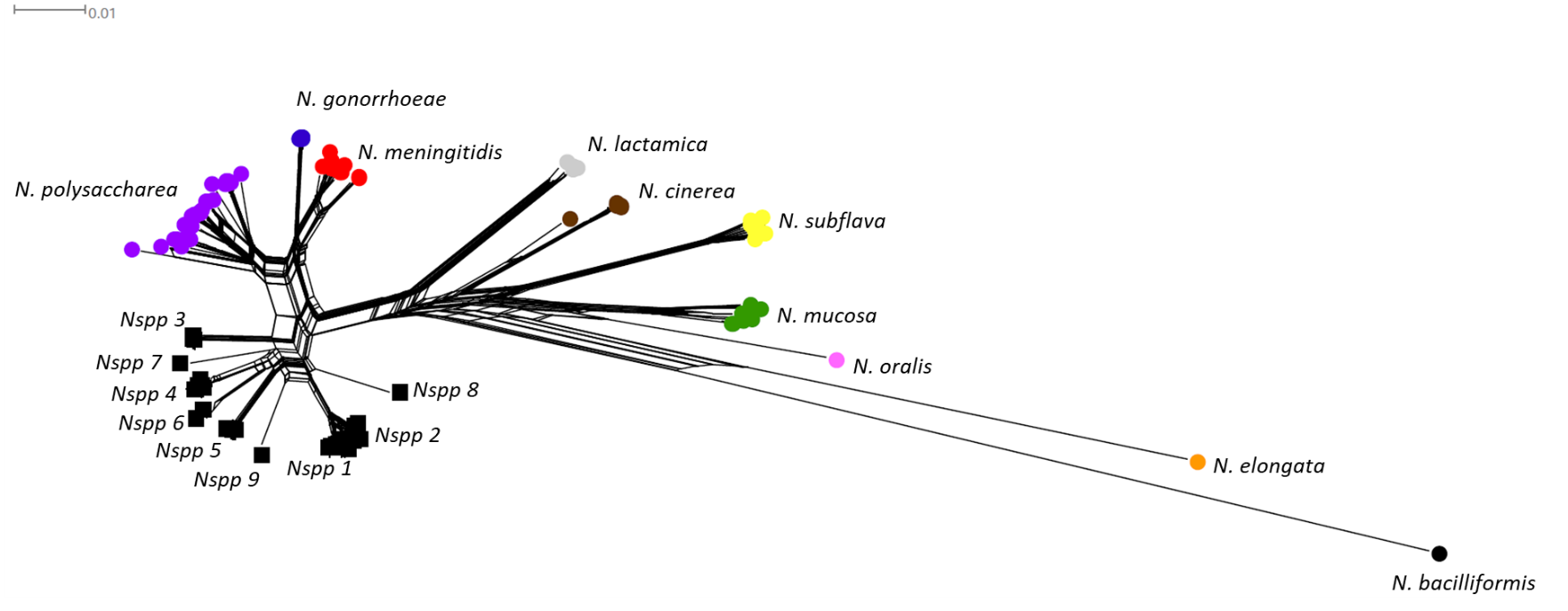


**Supplemental Figure 1: phylogenetic relationship of human-restricted *Neisseria* species.**

Neighbor-Net representation of the relationship between the new species and the 10 other human restricted *Neisseria* species, generated from the concatenated sequences of the complete 51 ribosomal loci.


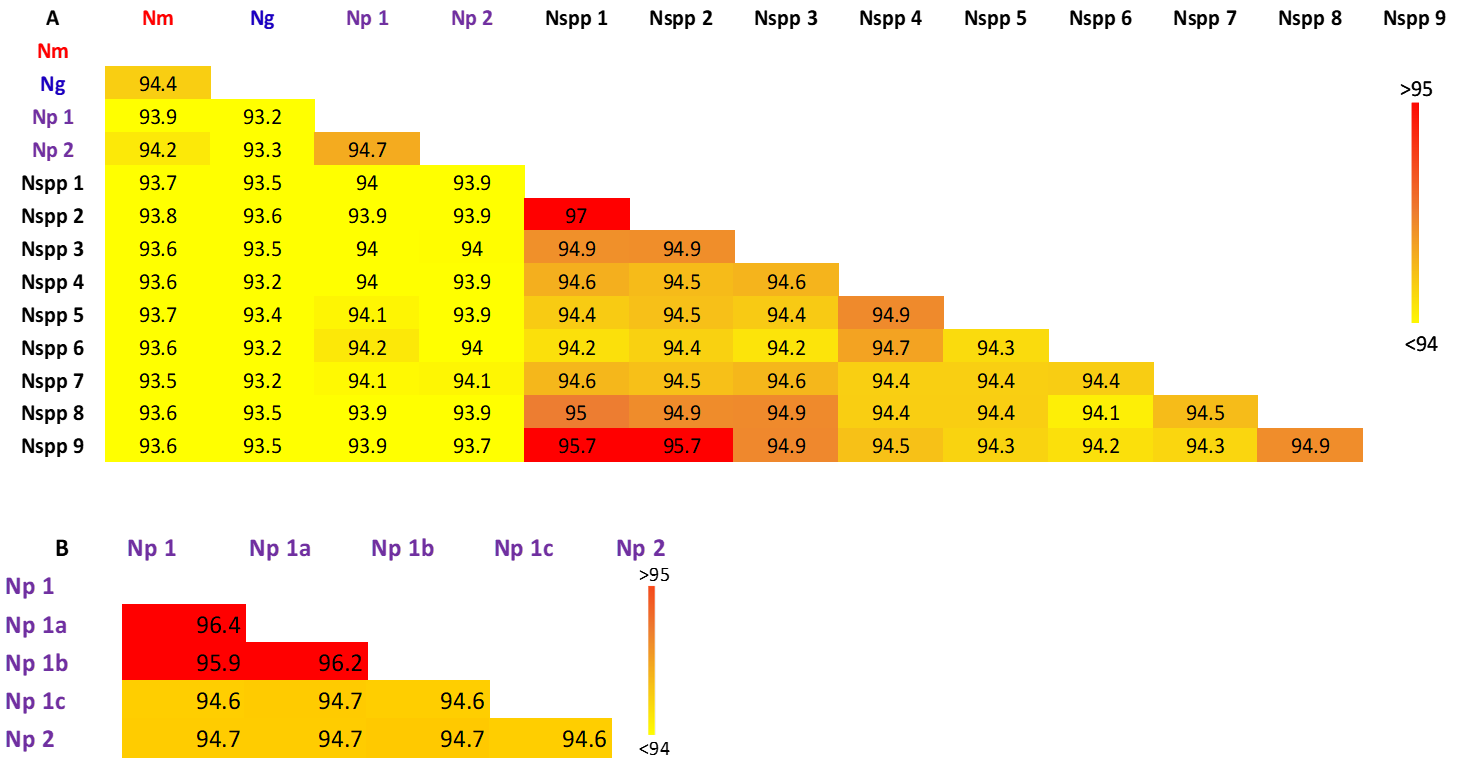


**Supplemental Figure 2:** **Heatmap of pairwise comparison of the whole genome sequences among the different clusters.** Two-way Average Nucleotide Identity using WGS among all the clusters (A) and only among the *N. polysaccharea* isolates (B). ANI measured in percentage are presented with a colour gradient, yellow for values <94 and red for values >95%, which is the threshold above which genomes are considered to be from the species. Nm: *N. meningitidis*; Ng: *N. gonorrhoeae*; Np: *N. polysaccharea* and Nspp: *Neisseria spp*


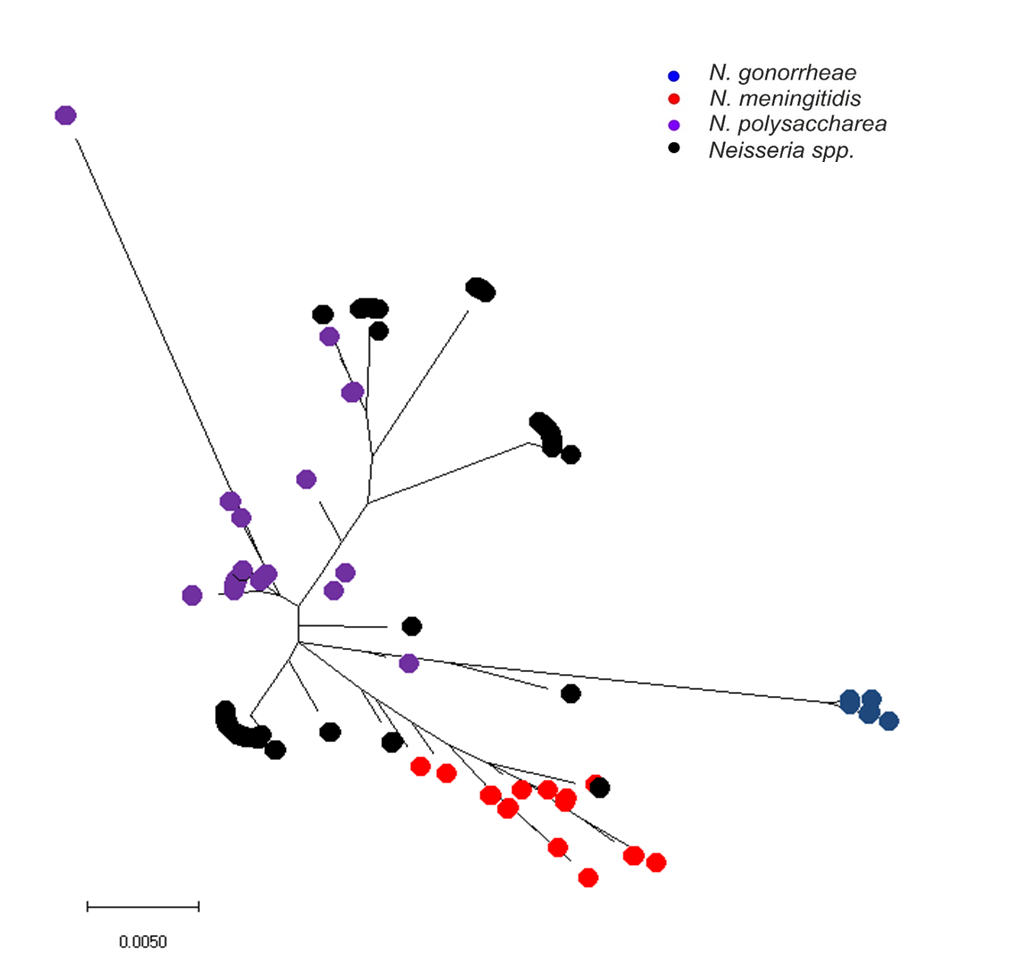


**Supplemental Figure 3:** 16s RNA Neighbour joining phylogeny of the 181 isolates included in this study.

**Supplemental Tables**

Supplemental Table 1: List of *Neisseria* isolates included in this study and additional meta-data associated with them, obtained from PubMLST

| \| PubMLST ID \| Isolate ID \| Country \| Year \| Disease \| Age  years \| Gender \| Clusters \| Reference ^a^ \| \| --- \| --- \| --- \| --- \| --- \| --- \| --- \| --- \| --- \| \| 2855 \| FA1090 ^§^ \| Unk \| Unk \| Unk \| Unk \| Unk \| Ng \| NC_002946 \| \| 13685 \| NCCP11945 \| South Korea \| Unk \| Unk \| Unk \| female \| Ng \| NC_011035 \| \| 21065 \| TCDC-NG08107 \| Unk \| 2008 \| Unk \| Unk \| Unk \| Ng \| Chen CC et al. 2011 \| \| 46272 \| 32867 \| Canada \| 2010 \| Unk \| Unk \| Unk \| Ng \| CP016015 \| \| 46273 \| 34530 \| Canada \| 2012 \| Unk \| Unk \| Unk \| Ng \| CP016016 \| \| 46274 \| 34769 \| Canada \| 2011 \| Unk \| Unk \| Unk \| Ng \| CP016017 \| \| 46275 \| FA19 \| Unk \| Unk \| Unk \| Unk \| Unk \| Ng \| Bennett JS et al. 2007 \| \| 46276 \| FA6140 \| USA \| Unk \| Unk \| Unk \| Unk \| Ng \| Bennett JS et al 2012 \| \| 46277 \| 12816 \| Sweden \| 2002 \| Unk \| Unk \| Unk \| Ng \| Abrams AJ et al 2015 \| \| 30 \| 14 \| Germany \| 1999 \| No \| 18 \| Unk \| Nm \| Claus H et al. 2005 \| \| 240 \| MC58 \| UK \| 1983 \| Unk \| Unk \| Unk \| Nm \| Tettelin H et al. 2000 \| \| 410 \| BZ 232 \| The Netherlands \| 1964 \| Yes \| Unk \| Unk \| Nm \| ERS006985 \| \| 613 \| Z2491 ^§^ \| The Gambia \| 1983 \| Yes \| Unk \| Unk \| Nm \| ERS006949 \| \| 638 \| G2136 \| UK \| 1986 \| Yes \| Unk \| Unk \| Nm \| ERS006919 \| \| 698 \| FAM18 \| USA \| 1983 \| Yes \| Unk \| Unk \| Nm \| Bentley SD et al. 2007 \| \| 1038 \| 8013 \| France \| 1989 \| Yes \| Unk \| Unk \| Nm \| Rusniok C et al. 2009 \| \| 19261 \| alpha710 \| Germany \| Unk \| No \| Unk \| Unk \| Nm \| Joseph B et al. 2010 \| \| 31318 \| NM3682 \| UK \| Unk \| Yes \| Unk \| Unk \| Nm \| CP009420 \| \| 34578 \| 88050 \| Chad \| 1988 \| Yes \| Unk \| Unk \| Nm \| NZ_ANRD00000000.1 \| \| 34607 \| 2004090 \| Niger \| 2004 \| Yes \| Unk \| Unk \| Nm \| NZ_ANRW00000000.1 \| \| 34610 \| 2007056 \| Burkina Faso \| 2007 \| Yes \| Unk \| Unk \| Nm \| NZ_ANSE00000000.1. \| \| 34655 \| NM3652 \| Bangladesh \| 2006 \| Yes \| Unk \| Unk \| Nm \| NZ_ANSC00000000.1 \| \| 36059 \| M22276 \| Mali \| 1969 \| Unk \| Unk \| Unk \| Nm \| Unpublished CDC- Retchless A. \| \| 38695 \| M22431 \| Burkina Faso \| 1978 \| Unk \| Unk \| Unk \| Nm \| Unpublished CDC- Retchless A. \| \| 39634 \| 583-15 \| Niger \| 2015 \| Unk \| Unk \| Unk \| Nm \| Unpublished, CDC- Retchless A. \| \| 39785 \| 1947-15 \| Niger \| 2015 \| Unk \| Unk \| Unk \| Nm \| Unpublished CDC- Retchless A. \| \| 39854 \| M05749 \| Norway \| 1987 \| Unk \| Unk \| Unk \| Nm \| Unpublished CDC- Retchless A. \| \| 39856 \| M05729 \| South Africa \| 1990 \| Unk \| Unk \| Unk \| Nm \| Unpublished CDC- Retchless A. \| \| 39860 \| M28679 \| UK \| 2014 \| Unk \| Unk \| Unk \| Nm \| Unpublished CDC- Retchless A. \| \| 19097 \| CCUG 24846 \| Unk \| Unk \| Unk \| Unk \| Unk \| Np 1 \| ERR026509 \| \| 19098 \| CCUG 27182 \| Unk \| Unk \| Unk \| Unk \| Unk \| Np 1 \| ERR026516 \| \| 21047 \| CCUG 4790 \| Unk \| Unk \| No \| Unk \| Unk \| Np 1 \| ERR160751 \| \| 36140 \| 12015_2014 \| Ireland \| 2014 \| No \| 3 \| Unk \| Np 1 \| ERR976832 \| \| 36142 \| 12017_2014 \| Ireland \| 2014 \| No \| 1 \| Unk \| Np 1 \| ERR976833 \| \| 36148 \| 12024_2014 \| Ireland \| 2014 \| No \| 4 \| Unk \| Np 1 \| ERR976838 \| \| 36153 \| 12030_2014 \| Ireland \| 2014 \| No \| 1 \| Unk \| Np 1 \| ERR976841 \| \| 36167 \| 12046_2014 \| Ireland \| 2014 \| No \| <1 \| Unk \| Np 1 \| ERR976854 \| \| 41652 \| 2748 ^Ŧ^ \| Italy \| 2015 \| Unk \| Unk \| Unk \| Np 1 \| Unpublished  Fazio C. \| \| 43987 \| LNP28609 \| France \| 2016 \| No \| Unk \| Unk \| Np 1 \| Unpublished  Taha MK, Hong E \| \| 45356 \| LNP28667 \| France \| 2016 \| No \| Unk \| Unk \| Np 1 \| Unpublished  Taha MK, Hong E \| \| 14730 \| ATCC 43768 \| Belgium \| Unk \| Unk \| Unk \| Unk \| Np 1/ Np 3 \| Marri PR et al. 2010 \| \| 19095 \| CCUG 18031* \| Germany \| 1984 \| No \| Unk \| Unk \| Np 1/ Np 3 \| ERR027247 \| \| 19096 \| CCUG 24845 \| Unk \| Unk \| No \| Unk \| Unk \| Np 1/ Np 3 \| ERR027248 \| \| 38980 \| LNP22706 \| France \| Unk \| Unk \| Unk \| Unk \| Np 1/ Np 3 \| Unpublished  Taha MK, Hong E \| \| 38981 \| LNP24574 \| France \| Unk \| Unk \| Unk \| Unk \| Np 1/ Np 3 \| Unpublished  Taha MK, Hong E \| \| 43420 \| 01_13564_XS3_1 \| Mali \| 2010 \| No \| 1 \| male \| Np 1/ Np 3 \| ERR1604226 \| \| 44084 \| ST41786 \| UK \| 2015 \| No \| 16 \| male \| Np 1/ Np 3 \| ERR1517321 \| \| 44102 \| BR40761 B \| UK \| 2014 \| No \| 16 \| male \| Np 1/ Np 3 \| ERR1517398 \| \| 46136 \| G97_6093 ^Ŧ^ \| The Gambia \| 1997 \| No \| 3 \| male \| Np 1/ Np 3 \| ERR1674672 \| \| 46200 \| G97_1061 \| The Gambia \| 1997 \| No \| 5 \| female \| Np 1/ Np 3 \| ERR1674736 \| \| 46409 \| M-080 \| Malawi \| 2005 \| No \| 2 \| female \| Np 1/ Np 3 \| ERR107381 \| \| 46417 \| M-104 \| Malawi \| 2005 \| No \| 5 \| female \| Np 1/ Np 3 \| ERR107389 \| \| 46418 \| M-104A \| Malawi \| 2005 \| No \| 5 \| female \| Np 1/ Np 3 \| ERR107390 \| \| 46428 \| M-198 \| Malawi \| 2005 \| No \| 5 \| female \| Np 1/ Np 3 \| ERR107400 \| \| 46437 \| M-223 *^§^ \| Malawi \| 2005 \| No \| 5 \| female \| Np 1/ Np 3 \| ERR107409 \| \| 46449 \| M-264 \| Malawi \| 2005 \| No \| 4 \| male \| Np 1/ Np 3 \| ERR107422 \| \| 46451 \| M-286 \| Malawi \| 2005 \| No \| 1 \| male \| Np 1/ Np 3 \| ERR107424 \| \| 46462 \| M-442 \| Malawi \| 2005 \| No \| <1 \| female \| Np 1/ Np 3 \| ERR107435 \| \| 46467 \| M-571 \| Malawi \| 2006 \| No \| 16 \| male \| Np 1/ Np 3 \| ERR107440 \| \| 46475 \| M-856 \| Malawi \| 2007 \| No \| 2 \| male \| Np 1/ Np 3 \| ERR107448 \| \| 46727 \| PL40288 *^Ŧ^ \| UK \| 2015 \| No \| 17 \| female \| Np 1/ Np 3 \| ERR1678065 \| \| 43335 \| 01_00098_XS1_1 \| Mali \| 2010 \| No \| 14 \| female \| Np 2 \| ERR1604112 \| \| 43413 \| 01_07665_XS1_1 \| Mali \| 2010 \| No \| 2 \| female \| Np 2 \| ERR1604219 \| \| 43415 \| 01_03967_XS1_1 \| Mali \| 2010 \| No \| 8 \| female \| Np 2 \| ERR1604221 \| \| 43416 \| 01_07343_XS1_1 \| Mali \| 2010 \| No \| 56 \| female \| Np 2 \| ERR1604222 \| \| 43417 \| 01_08009_XS1_1 \| Mali \| 2010 \| No \| 41 \| female \| Np 2 \| ERR1604223 \| \| 43418 \| 01_13553_XS3_1 \| Mali \| 2010 \| No \| <1 \| male \| Np 2 \| ERR1604224 \| \| 43419 \| 01_13554_XS3_1 \| Mali \| 2010 \| No \| 3 \| male \| Np 2 \| ERR1604225 \| \| 46143 \| G97_5907 \| The Gambia \| 1997 \| No \| 3 \| male \| Np 2 \| ERR1674679 \| \| 46173 \| G97_2571 *^§^ \| The Gambia \| 1997 \| No \| 4 \| male \| Np 2 \| ERR1674709 \| \| 46186 \| G97_1651 \| The Gambia \| 1997 \| No \| 3 \| male \| Np 2 \| ERR1674722 \| \| 46196 \| G97_1162 \| The Gambia \| 1997 \| No \| 4 \| male \| Np 2 \| ERR1674732 \| \| 46197 \| G97_1151 \| The Gambia \| 1997 \| No \| 4 \| male \| Np 2 \| ERR1674733 \| \| 46199 \| G97_1072 \| The Gambia \| 1997 \| No \| 4 \| male \| Np 2 \| ERR1674735 \| \| 42909 \| 02_11162_XS2_1 \| Mali \| 2011 \| No \| 8 \| female \| Nspp 1 \| ERR1603993 \| \| 43020 \| 02_11069_XS2_1 \| Mali \| 2011 \| No \| 12 \| male \| Nspp 1 \| ERR1730413 \| \| 43021 \| 02_11081_XS2_1 \| Mali \| 2011 \| No \| 5 \| female \| Nspp 1 \| ERR1730414 \| \| 43022 \| 02_11161_XS2_1 \| Mali \| 2011 \| No \| 2 \| female \| Nspp 1 \| ERR1730415 \| \| 43023 \| 02_11615_XS2_1 \| Mali \| 2011 \| No \| 21 \| male \| Nspp 1 \| ERR1730416 \| \| 43024 \| 02_11164_XS2_1 \| Mali \| 2011 \| No \| 2 \| female \| Nspp 1 \| ERR1730417 \| \| 43025 \| 02_11253_XS2_1 \| Mali \| 2011 \| No \| 2 \| female \| Nspp 1 \| ERR1730418 \| \| 43026 \| 02_11666_XS2_1 \| Mali \| 2011 \| No \| 17 \| male \| Nspp 1 \| ERR1730419 \| \| 43027 \| 02_11761_XS2_1 \| Mali \| 2011 \| No \| 34 \| female \| Nspp 1 \| ERR1730420 \| \| 43028 \| 02_11322_XS2_1 \| Mali \| 2011 \| No \| 4 \| male \| Nspp 1 \| ERR1730421 \| \| 43029 \| 02_11327_XS2_1 \| Mali \| 2011 \| No \| 14 \| male \| Nspp 1 \| ERR1730422 \| \| 43030 \| 02_11362_XS2_1 \| Mali \| 2011 \| No \| 7 \| male \| Nspp 1 \| ERR1730423 \| \| 43031 \| 02_11346_XS2_1 \| Mali \| 2011 \| No \| 4 \| female \| Nspp 1 \| ERR1730424 \| \| 43032 \| 02_11474_XS2_1 \| Mali \| 2011 \| No \| 3 \| female \| Nspp 1 \| ERR1730425 \| \| 43033 \| 02_11433_XS2_1 \| Mali \| 2011 \| No \| 5 \| male \| Nspp 1 \| ERR1730426 \| \| 43034 \| 02_13924_XS3_1 \| Mali \| 2012 \| No \| 6 \| male \| Nspp 1 \| ERR1730427 \| \| 43035 \| 02_13976_XS3_1 \| Mali \| 2012 \| No \| 11 \| male \| Nspp 1 \| ERR1730428 \| \| 43036 \| 02_14067_XS3_1 \| Mali \| 2012 \| No \| 4 \| male \| Nspp 1 \| ERR1730434 \| \| 43037 \| 02_13995_XS3_1 \| Mali \| 2012 \| No \| 11 \| female \| Nspp 1 \| ERR1730435 \| \| 43038 \| 02_14121_XS3_1 \| Mali \| 2012 \| No \| 6 \| male \| Nspp 1 \| ERR1730436 \| \| 43039 \| 01_13562_XS3_1 \| Mali \| 2012 \| No \| 7 \| female \| Nspp 1 \| ERR1730437 \| \| 43040 \| 01_13695_XS3_1 \| Mali \| 2012 \| No \| 6 \| female \| Nspp 1 \| ERR1730438 \| \| 43041 \| 01_13712_XS3_1 \| Mali \| 2012 \| No \| 5 \| male \| Nspp 1 \| ERR1730439 \| \| 43042 \| 01_12742_XS3_1 \| Mali \| 2012 \| No \| 23 \| female \| Nspp 1 \| ERR1730440 \| \| 43043 \| 01_13770_XS3_1 \| Mali \| 2012 \| No \| 13 \| male \| Nspp 1 \| ERR1730441 \| \| 43421 \| 01_00119_XS1_1 \| Mali \| 2010 \| No \| 11 \| male \| Nspp 1 \| ERR1730406 \| \| 43422 \| 01_03638_XS1_1 \| Mali \| 2010 \| No \| 2 \| female \| Nspp 1 \| ERR1730407 \| \| 43423 \| 01_03957_XS1_1* \| Mali \| 2010 \| No \| 9 \| male \| Nspp 1 \| ERR1730408 \| \| 43424 \| 02_01070_XS1_1 \| Mali \| 2010 \| No \| 7 \| male \| Nspp 1 \| ERR1730409 \| \| 43426 \| 02_01287_XS1_1*^§^ \| Mali \| 2010 \| No \| 41 \| female \| Nspp 1 \| ERR1730411 \| \| 43427 \| 02_07958_XS1_1 \| Mali \| 2010 \| No \| Unk \| Unknown \| Nspp 1 \| ERR1730412 \| \| 43428 \| 01_00007_XS1_1 \| Mali \| 2010 \| No \| 4 \| male \| Nspp 1 \| ERR1730429 \| \| 43429 \| 01_03581_XS1_1 \| Mali \| 2010 \| No \| 5 \| male \| Nspp 1 \| ERR1730430 \| \| 43430 \| 01_07472_XS1_1 \| Mali \| 2010 \| No \| 16 \| female \| Nspp 1 \| ERR1730431 \| \| 43431 \| 01_07795_XS1_1 \| Mali \| 2010 \| No \| 4 \| female \| Nspp 1 \| ERR1730432 \| \| 43432 \| 02_01039_XS1_1 \| Mali \| 2010 \| No \| 10 \| female \| Nspp 1 \| ERR1730433 \| \| 46230 \| M_676M \| Malawi \| 2006 \| No \| 22 \| female \| Nspp 2 \| ERR1674616 \| \| 46400 \| M-037 \| Malawi \| 2005 \| No \| 7 \| female \| Nspp 2 \| ERR107372 \| \| 46401 \| M-038 \| Malawi \| 2005 \| No \| 2 \| male \| Nspp 2 \| ERR107373 \| \| 46404 \| M-052 *^§^ \| Malawi \| 2005 \| No \| 4 \| female \| Nspp 2 \| ERR107376 \| \| 46406 \| M-065 \| Malawi \| 2005 \| No \| <1 \| male \| Nspp 2 \| ERR107378 \| \| 46421 \| M-129 \| Malawi \| 2005 \| No \| 4 \| male \| Nspp 2 \| ERR107393 \| \| 46422 \| M-130 \| Malawi \| 2005 \| No \| <1 \| male \| Nspp 2 \| ERR107394 \| \| 46424 \| M-161 \| Malawi \| 2005 \| No \| 11 \| male \| Nspp 2 \| ERR107396 \| \| 46427 \| M-190 \| Malawi \| 2005 \| No \| 7 \| female \| Nspp 2 \| ERR107399 \| \| 46434 \| M-214 \| Malawi \| 2005 \| No \| 5 \| female \| Nspp 2 \| ERR107406 \| \| 46443 \| M-250 \| Malawi \| 2005 \| No \| 11 \| female \| Nspp 2 \| ERR107415 \| \| 46444 \| M-252 \| Malawi \| 2005 \| No \| 8 \| female \| Nspp 2 \| ERR107416 \| \| 46445 \| M-256C * \| Malawi \| 2005 \| No \| 8 \| male \| Nspp 2 \| ERR107418 \| \| 46448 \| M-262 \| Malawi \| 2005 \| No \| 4 \| female \| Nspp 2 \| ERR107421 \| \| 46450 \| M-274 \| Malawi \| 2005 \| No \| 12 \| female \| Nspp 2 \| ERR107423 \| \| 46452 \| M-290 \| Malawi \| 2005 \| No \| 13 \| female \| Nspp 2 \| ERR107425 \| \| 46459 \| M-357 \| Malawi \| 2005 \| No \| 1 \| male \| Nspp 2 \| ERR107432 \| \| 46460 \| M-366 \| Malawi \| 2005 \| No \| <1 \| female \| Nspp 2 \| ERR107433 \| \| 46469 \| M-718M \| Malawi \| 2007 \| No \| 21 \| female \| Nspp 2 \| ERR107442 \| \| 46470 \| M-760 \| Malawi \| 2007 \| No \| 13 \| male \| Nspp 2 \| ERR107443 \| \| 43128 \| 12_12795_XS2_1 \| Chad \| 2011 \| No \| 9 \| female \| Nspp 3 \| ERR1730442 \| \| 43129 \| 12_13955_XS2_1* \| Chad \| 2011 \| No \| 10 \| female \| Nspp 3 \| ERR1730443 \| \| 43130 \| 12_14141_XS2_1 \| Chad \| 2011 \| No \| 1 \| female \| Nspp 3 \| ERR1730444 \| \| 43131 \| 12_23282_XS3_1 \| Chad \| 2011 \| No \| 6 \| male \| Nspp 3 \| ERR1730445 \| \| 43132 \| 12_13996_XS2_1 \| Chad \| 2011 \| No \| 4 \| female \| Nspp 3 \| ERR1730446 \| \| 43133 \| 12_13835_XS2_1 \| Chad \| 2011 \| No \| 12 \| female \| Nspp 3 \| ERR1730447 \| \| 43134 \| 12_13988_XS2_1*^§^ \| Chad \| 2011 \| No \| 6 \| male \| Nspp 3 \| ERR1730448 \| \| 43135 \| 12_17465_XS2_1 \| Chad \| 2011 \| No \| 1 \| male \| Nspp 3 \| ERR1730449 \| \| 43136 \| 12_14600_XS2_1 \| Chad \| 2011 \| No \| 4 \| female \| Nspp 3 \| ERR1730450 \| \| 46231 \| M_700M \| Malawi \| 2006 \| No \| 32 \| female \| Nspp 4 \| ERR1674617 \| \| 46402 \| M-041 * \| Malawi \| 2005 \| No \| 1 \| male \| Nspp 4 \| ERR107374 \| \| 46403 \| M-046 \| Malawi \| 2005 \| No \| <1 \| female \| Nspp 4 \| ERR107375 \| \| 46410 \| M-089 \| Malawi \| 2005 \| No \| 9 \| female \| Nspp 4 \| ERR107382 \| \| 46423 \| M-131 \| Malawi \| 2005 \| No \| 1 \| male \| Nspp 4 \| ERR107395 \| \| 46425 \| M-173 \| Malawi \| 2005 \| No \| 2 \| male \| Nspp 4 \| ERR107397 \| \| 46426 \| M-174 \| Malawi \| 2005 \| No \| 4 \| male \| Nspp 4 \| ERR107398 \| \| 46435 \| M-216 \| Malawi \| 2005 \| No \| 7 \| female \| Nspp 4 \| ERR107407 \| \| 46441 \| M-244 \| Malawi \| 2005 \| No \| 8 \| male \| Nspp 4 \| ERR107413 \| \| 46447 \| M-261 \| Malawi \| 2005 \| No \| 3 \| female \| Nspp 4 \| ERR107420 \| \| 46455 \| M-318 \| Malawi \| 2005 \| No \| 2 \| male \| Nspp 4 \| ERR107428 \| \| 46456 \| M-337 \| Malawi \| 2005 \| No \| 2 \| male \| Nspp 4 \| ERR107429 \| \| 46457 \| M-338 *^§^ \| Malawi \| 2005 \| No \| <1 \| female \| Nspp 4 \| ERR107430 \| \| 46458 \| M-345 \| Malawi \| 2005 \| No \| 1 \| male \| Nspp 4 \| ERR107431 \| \| 46405 \| M-064 \| Malawi \| 2005 \| No \| 2 \| male \| Nspp 5 \| ERR107377 \| \| 46408 \| M-079 \| Malawi \| 2005 \| No \| 4 \| female \| Nspp 5 \| ERR107380 \| \| 46415 \| M-101 *^§^ \| Malawi \| 2005 \| No \| 1 \| female \| Nspp 5 \| ERR107387 \| \| 46416 \| M-102 \| Malawi \| 2005 \| No \| 4 \| male \| Nspp 5 \| ERR107388 \| \| 46429 \| M-200 \| Malawi \| 2005 \| No \| 3 \| male \| Nspp 5 \| ERR107401 \| \| 46431 \| M-210 \| Malawi \| 2005 \| No \| 4 \| male \| Nspp 5 \| ERR107403 \| \| 46432 \| M-211 \| Malawi \| 2005 \| No \| 11 \| female \| Nspp 5 \| ERR107404 \| \| 46438 \| M-225 \| Malawi \| 2005 \| No \| 6 \| female \| Nspp 5 \| ERR107410 \| \| 46439 \| M-226 * \| Malawi \| 2005 \| No \| 9 \| male \| Nspp 5 \| ERR107411 \| \| 46442 \| M-249 \| Malawi \| 2005 \| No \| 13 \| female \| Nspp 5 \| ERR107414 \| \| 46461 \| M-384 \| Malawi \| 2005 \| No \| 1 \| male \| Nspp 5 \| ERR107434 \| \| 46463 \| M-448 \| Malawi \| 2005 \| No \| 1 \| male \| Nspp 5 \| ERR107436 \| \| 46464 \| M-456 \| Malawi \| 2005 \| No \| 1 \| female \| Nspp 5 \| ERR107437 \| \| 46465 \| M-457 \| Malawi \| 2005 \| No \| 1 \| male \| Nspp 5 \| ERR107438 \| \| 46468 \| M-672M \| Malawi \| 2006 \| No \| 23 \| female \| Nspp 5 \| ERR107441 \| \| 46471 \| M-784 \| Malawi \| 2007 \| No \| 7 \| male \| Nspp 5 \| ERR107444 \| \| 46472 \| M-786 \| Malawi \| 2007 \| No \| 3 \| male \| Nspp 5 \| ERR107445 \| \| 46473 \| M-802 \| Malawi \| 2007 \| No \| 5 \| male \| Nspp 5 \| ERR107446 \| \| 46476 \| M-872 \| Malawi \| 2007 \| No \| 2 \| male \| Nspp 5 \| ERR107449 \| \| 46477 \| M-875 \| Malawi \| 2007 \| No \| 2 \| male \| Nspp 5 \| ERR107450 \| \| 46478 \| M-879 \| Malawi \| 2007 \| No \| 1 \| female \| Nspp 5 \| ERR107451 \| \| 44518 \| OX40911 * \| UK \| 2015 \| No \| 16 \| female \| Nspp 6 \| ERR1600478 \| \| 46411 \| M-091 *^§^ \| Malawi \| 2005 \| No \| 1 \| male \| Nspp 6 \| ERR107383 \| \| 46412 \| M-092 \| Malawi \| 2005 \| No \| 4 \| female \| Nspp 6 \| ERR107384 \| \| 46192 \| G97_1403 *^§^ \| The Gambia \| 1997 \| No \| 4 \| male \| Nspp 7 \| ERR1674728 \| \| 46193 \| G97_1402 \| The Gambia \| 1997 \| No \| 4 \| male \| Nspp 7 \| ERR1674729 \| \| 43425 \| 02_01105_XS1_1*^§^ \| Mali \| 2010 \| No \| 8 \| female \| Nspp 8 \| ERR1730410 \| \| 5193 \| 15883 *^§^ \| Germany \| 1984 \| No \| 2 \| Unk \| Nspp 9 \| ERR028376 \| |
| --- | --- | --- | --- | --- | --- | --- | --- | --- | --- | --- | --- | --- | --- | --- | --- | --- | --- | --- | --- | --- | --- | --- | --- | --- | --- | --- | --- | --- | --- | --- | --- | --- | --- | --- | --- | --- | --- | --- | --- | --- | --- | --- | --- | --- | --- | --- | --- | --- | --- | --- | --- | --- | --- | --- | --- | --- | --- | --- | --- | --- | --- | --- | --- | --- | --- | --- | --- | --- | --- | --- | --- | --- | --- | --- | --- | --- | --- | --- | --- | --- | --- | --- | --- | --- | --- | --- | --- | --- | --- | --- | --- | --- | --- | --- | --- | --- | --- | --- | --- | --- | --- | --- | --- | --- | --- | --- | --- | --- | --- | --- | --- | --- | --- | --- | --- | --- | --- | --- | --- | --- | --- | --- | --- | --- | --- | --- | --- | --- | --- | --- | --- | --- | --- | --- | --- | --- | --- | --- | --- | --- | --- | --- | --- | --- | --- | --- | --- | --- | --- | --- | --- | --- | --- | --- | --- | --- | --- | --- | --- | --- | --- | --- | --- | --- | --- | --- | --- | --- | --- | --- | --- | --- | --- | --- | --- | --- | --- | --- | --- | --- | --- | --- | --- | --- | --- | --- | --- | --- | --- | --- | --- | --- | --- | --- | --- | --- | --- | --- | --- | --- | --- | --- | --- | --- | --- | --- | --- | --- | --- | --- | --- | --- | --- | --- | --- | --- | --- | --- | --- | --- | --- | --- | --- | --- | --- | --- | --- | --- | --- | --- | --- | --- | --- | --- | --- | --- | --- | --- | --- | --- | --- | --- | --- | --- | --- | --- | --- | --- | --- | --- | --- | --- | --- | --- | --- | --- | --- | --- | --- | --- | --- | --- | --- | --- | --- | --- | --- | --- | --- | --- | --- | --- | --- | --- | --- | --- | --- | --- | --- | --- | --- | --- | --- | --- | --- | --- | --- | --- | --- | --- | --- | --- | --- | --- | --- | --- | --- | --- | --- | --- | --- | --- | --- | --- | --- | --- | --- | --- | --- | --- | --- | --- | --- | --- | --- | --- | --- | --- | --- | --- | --- | --- | --- | --- | --- | --- | --- | --- | --- | --- | --- | --- | --- | --- | --- | --- | --- | --- | --- | --- | --- | --- | --- | --- | --- | --- | --- | --- | --- | --- | --- | --- | --- | --- | --- | --- | --- | --- | --- | --- | --- | --- | --- | --- | --- | --- | --- | --- | --- | --- | --- | --- | --- | --- | --- | --- | --- | --- | --- | --- | --- | --- | --- | --- | --- | --- | --- | --- | --- | --- | --- | --- | --- | --- | --- | --- | --- | --- | --- | --- | --- | --- | --- | --- | --- | --- | --- | --- | --- | --- | --- | --- | --- | --- | --- | --- | --- | --- | --- | --- | --- | --- | --- | --- | --- | --- | --- | --- | --- | --- | --- | --- | --- | --- | --- | --- | --- | --- | --- | --- | --- | --- | --- | --- | --- | --- | --- | --- | --- | --- | --- | --- | --- | --- | --- | --- | --- | --- | --- | --- | --- | --- | --- | --- | --- | --- | --- | --- | --- | --- | --- | --- | --- | --- | --- | --- | --- | --- | --- | --- | --- | --- | --- | --- | --- | --- | --- | --- | --- | --- | --- | --- | --- | --- | --- | --- | --- | --- | --- | --- | --- | --- | --- | --- | --- | --- | --- | --- | --- | --- | --- | --- | --- | --- | --- | --- | --- | --- | --- | --- | --- | --- | --- | --- | --- | --- | --- | --- | --- | --- | --- | --- | --- | --- | --- | --- | --- | --- | --- | --- | --- | --- | --- | --- | --- | --- | --- | --- | --- | --- | --- | --- | --- | --- | --- | --- | --- | --- | --- | --- | --- | --- | --- | --- | --- | --- | --- | --- | --- | --- | --- | --- | --- | --- | --- | --- | --- | --- | --- | --- | --- | --- | --- | --- | --- | --- | --- | --- | --- | --- | --- | --- | --- | --- | --- | --- | --- | --- | --- | --- | --- | --- | --- | --- | --- | --- | --- | --- | --- | --- | --- | --- | --- | --- | --- | --- | --- | --- | --- | --- | --- | --- | --- | --- | --- | --- | --- | --- | --- | --- | --- | --- | --- | --- | --- | --- | --- | --- | --- | --- | --- | --- | --- | --- | --- | --- | --- | --- | --- | --- | --- | --- | --- | --- | --- | --- | --- | --- | --- | --- | --- | --- | --- | --- | --- | --- | --- | --- | --- | --- | --- | --- | --- | --- | --- | --- | --- | --- | --- | --- | --- | --- | --- | --- | --- | --- | --- | --- | --- | --- | --- | --- | --- | --- | --- | --- | --- | --- | --- | --- | --- | --- | --- | --- | --- | --- | --- | --- | --- | --- | --- | --- | --- | --- | --- | --- | --- | --- | --- | --- | --- | --- | --- | --- | --- | --- | --- | --- | --- | --- | --- | --- | --- | --- | --- | --- | --- | --- | --- | --- | --- | --- | --- | --- | --- | --- | --- | --- | --- | --- | --- | --- | --- | --- | --- | --- | --- | --- | --- | --- | --- | --- | --- | --- | --- | --- | --- | --- | --- | --- | --- | --- | --- | --- | --- | --- | --- | --- | --- | --- | --- | --- | --- | --- | --- | --- | --- | --- | --- | --- | --- | --- | --- | --- | --- | --- | --- | --- | --- | --- | --- | --- | --- | --- | --- | --- | --- | --- | --- | --- | --- | --- | --- | --- | --- | --- | --- | --- | --- | --- | --- | --- | --- | --- | --- | --- | --- | --- | --- | --- | --- | --- | --- | --- | --- | --- | --- | --- | --- | --- | --- | --- | --- | --- | --- | --- | --- | --- | --- | --- | --- | --- | --- | --- | --- | --- | --- | --- | --- | --- | --- | --- | --- | --- | --- | --- | --- | --- | --- | --- | --- | --- | --- | --- | --- | --- | --- | --- | --- | --- | --- | --- | --- | --- | --- | --- | --- | --- | --- | --- | --- | --- | --- | --- | --- | --- | --- | --- | --- | --- | --- | --- | --- | --- | --- | --- | --- | --- | --- | --- | --- | --- | --- | --- | --- | --- | --- | --- | --- | --- | --- | --- | --- | --- | --- | --- | --- | --- | --- | --- | --- | --- | --- | --- | --- | --- | --- | --- | --- | --- | --- | --- | --- | --- | --- | --- | --- | --- | --- | --- | --- | --- | --- | --- | --- | --- | --- | --- | --- | --- | --- | --- | --- | --- | --- | --- | --- | --- | --- | --- | --- | --- | --- | --- | --- | --- | --- | --- | --- | --- | --- | --- | --- | --- | --- | --- | --- | --- | --- | --- | --- | --- | --- | --- | --- | --- | --- | --- | --- | --- | --- | --- | --- | --- | --- | --- | --- | --- | --- | --- | --- | --- | --- | --- | --- | --- | --- | --- | --- | --- | --- | --- | --- | --- | --- | --- | --- | --- | --- | --- | --- | --- | --- | --- | --- | --- | --- | --- | --- | --- | --- | --- | --- | --- | --- | --- | --- | --- | --- | --- | --- | --- | --- | --- | --- | --- | --- | --- | --- | --- | --- | --- | --- | --- | --- | --- | --- | --- | --- | --- | --- | --- | --- | --- | --- | --- | --- | --- | --- | --- | --- | --- | --- | --- | --- | --- | --- | --- | --- | --- | --- | --- | --- | --- | --- | --- | --- | --- | --- | --- | --- | --- | --- | --- | --- | --- | --- | --- | --- | --- | --- | --- | --- | --- | --- | --- | --- | --- | --- | --- | --- | --- | --- | --- | --- | --- | --- | --- | --- | --- | --- | --- | --- | --- | --- | --- | --- | --- | --- | --- | --- | --- | --- | --- | --- | --- | --- | --- | --- | --- | --- | --- | --- | --- | --- | --- | --- | --- | --- | --- | --- | --- | --- | --- | --- | --- | --- | --- | --- | --- | --- | --- | --- | --- | --- | --- | --- | --- | --- | --- | --- | --- | --- | --- | --- | --- | --- | --- | --- | --- | --- | --- | --- | --- | --- | --- | --- | --- | --- | --- | --- | --- | --- | --- | --- | --- | --- | --- | --- | --- | --- | --- | --- | --- | --- | --- | --- | --- | --- | --- | --- | --- | --- | --- | --- | --- | --- | --- | --- | --- | --- | --- | --- | --- | --- | --- | --- | --- | --- | --- | --- | --- | --- | --- | --- | --- | --- | --- | --- | --- | --- | --- | --- | --- | --- | --- | --- | --- | --- | --- | --- | --- | --- | --- | --- | --- | --- | --- | --- | --- | --- | --- | --- | --- | --- | --- | --- | --- | --- | --- | --- | --- | --- | --- | --- | --- | --- | --- | --- | --- | --- | --- | --- | --- | --- | --- | --- | --- | --- | --- | --- | --- | --- | --- | --- | --- | --- | --- | --- | --- | --- | --- | --- | --- | --- | --- | --- | --- | --- | --- | --- | --- | --- | --- | --- | --- | --- | --- | --- | --- | --- | --- | --- | --- | --- | --- | --- | --- | --- | --- | --- | --- | --- | --- | --- | --- | --- | --- | --- | --- | --- | --- | --- | --- | --- | --- | --- | --- | --- | --- | --- | --- | --- | --- | --- | --- | --- | --- | --- | --- | --- | --- | --- | --- | --- | --- | --- | --- | --- | --- | --- | --- | --- | --- | --- | --- | --- | --- | --- | --- | --- | --- | --- | --- | --- | --- | --- | --- | --- | --- | --- | --- | --- | --- | --- | --- | --- | --- | --- | --- | --- | --- | --- | --- | --- | --- | --- | --- | --- | --- | --- | --- | --- | --- | --- | --- | --- | --- | --- | --- | --- | --- | --- | --- | --- | --- | --- | --- | --- | --- | --- | --- | --- | --- | --- | --- | --- | --- | --- | --- | --- | --- | --- | --- | --- | --- | --- | --- | --- | --- | --- | --- | --- | --- | --- | --- | --- | --- | --- | --- | --- | --- | --- | --- | --- | --- | --- | --- | --- | --- | --- | --- | --- | --- | --- | --- | --- | --- | --- | --- | --- | --- | --- | --- | --- | --- | --- | --- | --- | --- | --- | --- | --- | --- | --- | --- | --- | --- | --- | --- | --- | --- | --- | --- | --- | --- | --- | --- | --- | --- | --- | --- | --- | --- | --- | --- | --- | --- | --- | --- | --- | --- | --- | --- | --- | --- | --- | --- | --- | --- | --- | --- | --- | --- | --- | --- | --- | --- | --- | --- | --- | --- | --- | --- | --- | --- | --- | --- | --- | --- | --- | --- | --- | --- | --- | --- | --- | --- | --- | --- | --- | --- | --- | --- | --- | --- | --- | --- | --- | --- | --- | --- | --- | --- | --- | --- | --- | --- | --- | --- | --- | --- | --- | --- | --- | --- | --- | --- | --- | --- | --- | --- | --- | --- | --- | --- | --- | --- | --- | --- | --- | --- | --- | --- | --- | --- | --- | --- | --- | --- | --- | --- | --- | --- | --- | --- | --- | --- | --- | --- | --- | --- | --- | --- | --- | --- | --- | --- |

*Nm*: *N. meningitidis*; *Ng*: *N. gonorrhoeae*; *Np*: *N. polysaccharea* and *Nspp*: *Neisseria spp*; Unk: Unknown

* Isolates used for the phenotypic analysis

^§^ Isolates used for the first ANI analysis

^Ŧ^ additional *Np* 1 isolates for second ANI analysis

^a^ ENA, GenBank Accession number or publication when available

Supplemental Table 2: cgMLST list of genes and probable function

| Locus | Full name | sequence status |
| --- | --- | --- |
| NEIS0001 | UDP-3-O-[3-hydroxymyristoyl] N-acetylglucosamine deacetylase (EC 3.5.1.-) | Complete |
| NEIS0004 | peptidyl-prolyl cis-trans isomerase | Complete |
| NEIS0006 | glycerate dehydrogenase | Complete |
| NEIS0008 | glucosamine--fructose-6-phosphate aminotransferase | Complete |
| NEIS0010 | outer membrane lipoprotein Gna33 | Complete |
| NEIS0011 | hypothetical protein | Complete |
| NEIS0012 | hypothetical protein | Complete |
| NEIS0013 | hypothetical protein | Complete |
| NEIS0014 | putative phosphonoacetate hydrolase | Complete |
| NEIS0015 | UDP-N-acetylglucosamine pyrophosphorylase | Complete |
| NEIS0017 | putative solute-binding periplasmic protein | Complete |
| NEIS0018 | putative inner membrane protein | Complete |
| NEIS0019 | hypothetical protein | Complete |
| NEIS0020 | peptide methionine sulfoxide reductase MsrA/MsrB | Incomplete |
| NEIS0021 | probable signal recognition particle protein cell division protein | Incomplete |
| NEIS0034 | putative inner membrane protein | Incomplete |
| NEIS0035 | PilT-like protein | Complete |
| NEIS0036 | type IV pilus retraction ATPase PilT | Complete |
| NEIS0037 | hypothetical protein | Incomplete |
| NEIS0038 | putative lipoprotein | Complete |
| NEIS0039 | pyrroline-5-carboxylate reductase | Incomplete |
| NEIS0040 | DnaK suppressor protein | Complete |
| NEIS0043 | chaperone protein DnaJ | Complete |
| NEIS0045 | dTDP-4-dehydrorhamnose 3,5-epimerase | Incomplete |
| NEIS0048 | UDP-glucose epimerase | Incomplete |
| NEIS0059 | putative transcriptional accessory protein | Incomplete |
| NEIS0069 | sodium/glutamate symport carrier protein | Complete |
| NEIS0071 | putative lipoprotein | Complete |
| NEIS0073 | outer membrane transport protein | Complete |
| NEIS0074 | pyruvate kinase (EC 2.7.1.40) | Complete |
| NEIS0092 | putative inner membrane protein | Complete |
| NEIS0097 | hypothetical protein | Incomplete |
| NEIS0098 | aspartate carbamoyltransferase catalytic subunit | Complete |
| NEIS0099 | pyrI | Complete |
| NEIS0100 | hypothetical protein | Complete |
| NEIS0101 | hypothetical protein | Complete |
| NEIS0102 | peptide deformylase | Complete |
| NEIS0103 | methionyl-tRNA formyltransferase | Complete |
| NEIS0104 | SUN-family protein | Incomplete |
| NEIS0105 | hypothetical protein | Incomplete |
| NEIS0106 | putative two-component sensor kinase | Incomplete |
| NEIS0107 | putative two-component trancriptional regulator | Complete |
| NEIS0108 | SMF-family protein | Complete |
| NEIS0109 | SMG-family protein | Complete |
| NEIS0110 | DNA topoisomerase I | Complete |
| NEIS0113 | hypothetical protein | Complete |
| NEIS0114 | hypothetical protein | Complete |
| NEIS0115 | putative ferredoxin | Complete |
| NEIS0118 | transcription antitermination protein | Complete |
| NEIS0119 | 50S ribosomal protein L11 | Complete |
| NEIS0120 | 50S ribosomal protein L1 | Complete |
| NEIS0121 | 50S ribosomal protein L10 | Complete |
| NEIS0122 | 50S ribosomal protein L7/L12 | Complete |
| NEIS0123 | DNA-directed RNA polymerase subunit beta | Incomplete |
| NEIS0124 | DNA-directed RNA polymerase subunit beta' | Incomplete |
| NEIS0125 | 30S ribosomal protein S12 | Complete |
| NEIS0126 | 30S ribosomal protein S7 | Complete |
| NEIS0127 | elongation factor G | Complete |
| NEIS0129 | 30S ribosomal protein S10 | Complete |
| NEIS0132 | 50S ribosomal protein L3 | Complete |
| NEIS0133 | 50S ribosomal protein L4 | Complete |
| NEIS0134 | 50S ribosomal protein L23 | Complete |
| NEIS0135 | 50S ribosomal protein L2 | Complete |
| NEIS0136 | 30S ribosomal protein S19 | Complete |
| NEIS0137 | 50S ribosomal protein L22 | Complete |
| NEIS0138 | 30S ribosomal protein S3 | Complete |
| NEIS0140 | 50S ribosomal protein L29 | Complete |
| NEIS0141 | 30S ribosomal protein S17 | Complete |
| NEIS0143 | 50S ribosomal protein L24 | Complete |
| NEIS0144 | 50S ribosomal protein L5 | Complete |
| NEIS0145 | 30S ribosomal protein S14 | Complete |
| NEIS0146 | 30S ribosomal protein S8 | Complete |
| NEIS0147 | 50S ribosomal protein L6 | Complete |
| NEIS0149 | 30S ribosomal protein S5 | Complete |
| NEIS0150 | 50S ribosomal protein L30 | Complete |
| NEIS0152 | preprotein translocase subunit SecY | Complete |
| NEIS0155 | 30S ribosomal protein S13 | Complete |
| NEIS0157 | 30S ribosomal protein S4 | Complete |
| NEIS0158 | DNA-directed RNA polymerase subunit alpha | Complete |
| NEIS0159 | 50S ribosomal protein L17 | Complete |
| NEIS0160 | septum formation inhibitor | Complete |
| NEIS0161 | septum site-determining protein | Complete |
| NEIS0162 | cell division topological specificity factor MinE | Complete |
| NEIS0163 | putative hydrogen peroxide-inducible genes activator | Incomplete |
| NEIS0164 | valyl-tRNA synthetase | Incomplete |
| NEIS0168 | UDP-N-acetylglucosamine acyltransferase | Complete |
| NEIS0170 | (3R)-hydroxymyristoyl-ACP dehydratase | Complete |
| NEIS0172 | putative outer membrane protein | Complete |
| NEIS0173 | outer membrane protein OMP85 | Complete |
| NEIS0174 | putative inner membrane protease | Incomplete |
| NEIS0175 | 1-deoxy-D-xylulose 5-phosphate reductoisomerase | Complete |
| NEIS0176 | phosphatidate cytidylyltransferase | Incomplete |
| NEIS0177 | putative undecaprenyl diphosphate synthase | Complete |
| NEIS0178 | ribosome recycling factor | Complete |
| NEIS0179 | putative inner membrane protein | Incomplete |
| NEIS0180 | hypothetical protein | Incomplete |
| NEIS0181 | 16S rRNA methyltransferase GidB | Complete |
| NEIS0182 | hypothetical protein | Complete |
| NEIS0183 | ribonuclease HII | Complete |
| NEIS0184 | tRNA uridine 5-carboxymethylaminomethyl modification enzyme GidA | Incomplete |
| NEIS0185 | putative inner membrane amino-acid transport protein | Complete |
| NEIS0186 | 4-hydroxythreonine-4-phosphate dehydrogenase | Incomplete |
| NEIS0187 | ribonuclease E | Incomplete |
| NEIS0190 | ribosomal large subunit pseudouridine synthase C | Complete |
| NEIS0191 | lipid-A-disaccharide synthase (EC 2.4.1.182) | Incomplete |
| NEIS0195 | dihydrodipicolinate reductase | Complete |
| NEIS0196 | putative outer membrane lipoprotein | Complete |
| NEIS0197 | ferric uptake regulation protein | Incomplete |
| NEIS0198 | leucyl/phenylalanyl-tRNA--protein transferase | Complete |
| NEIS0199 | glyceraldehyde 3-phosphate dehydrogenase (EC 1.2.1.12) | Complete |
| NEIS0200 | putative ferredoxin | Incomplete |
| NEIS0201 | putative inner membrane transport protein | Complete |
| NEIS0204 | DNA gyrase subunit B | Complete |
| NEIS0205 | putative inner membrane protein | Incomplete |
| NEIS0206 | oligopeptidase A | Complete |
| NEIS0207 | conserved hypothetical protein | Complete |
| NEIS0211 | catalase (KatA) | Incomplete |
| NEIS0216 | 3-oxoacyl-(acyl carrier protein) synthase II | Complete |
| NEIS0217 | acyl carrier protein | Complete |
| NEIS0218 | dihydroorotate dehydrogenase 2 | Complete |
| NEIS0222 | hypothetical protein | Complete |
| NEIS0224 | bifunctional glutamine-synthetase adenylyltransferase/deadenyltransferase | Incomplete |
| NEIS0230 | DNA helicase II | Incomplete |
| NEIS0237 | NADH dehydrogenase I chain A | Complete |
| NEIS0238 | nuoB; NADH dehydrogenase subunit B | Incomplete |
| NEIS0239 | NADH dehydrogenase subunit C | Incomplete |
| NEIS0240 | NADH dehydrogenase subunit D | Complete |
| NEIS0241 | NADH dehydrogenase subunit E | Complete |
| NEIS0242 | NADH dehydrogenase I chain F | Complete |
| NEIS0244 | NADH dehydrogenase subunit G | Complete |
| NEIS0245 | NADH dehydrogenase I chain H | Complete |
| NEIS0246 | NADH dehydrogenase subunit I | Complete |
| NEIS0247 | NADH dehydrogenase I chain J | Complete |
| NEIS0248 | NADH dehydrogenase subunit K | Complete |
| NEIS0251 | NADH dehydrogenase subunit L | Complete |
| NEIS0252 | NADH dehydrogenase subunit M | Complete |
| NEIS0253 | NADH dehydrogenase subunit N | Incomplete |
| NEIS0254 | inner membrane protein | Complete |
| NEIS0255 | geranyltranstransferase | Complete |
| NEIS0256 | exodeoxyribonuclease VII small subunit | Complete |
| NEIS0257 | ribosome-associated GTPase | Complete |
| NEIS0258 | putative ABC transporter | Complete |
| NEIS0259 | Holliday junction DNA helicase RuvA | Complete |
| NEIS0260 | hypothetical protein | Complete |
| NEIS0261 | putative periplasmic protein | Complete |
| NEIS0262 | putative tRNA/rRNA methyltransferase | Complete |
| NEIS0263 | hypothetical protein | Complete |
| NEIS0264 | hydrolase | Complete |
| NEIS0265 | hypothetical protein | Complete |
| NEIS0269 | ATP-dependent DNA helicase | Incomplete |
| NEIS0270 | indole-3-glycerol-phosphate synthase | Incomplete |
| NEIS0271 | hypothetical protein | Complete |
| NEIS0272 | putative inner membrane protein | Complete |
| NEIS0273 | thiol:disulphide interchange protein encodes DsbA1; oxidoreductase | Complete |
| NEIS0274 | hypothetical protein | Complete |
| NEIS0275 | putative outer membrane solvent tolerance protein | Complete |
| NEIS0276 | putative rotamase | Complete |
| NEIS0288 | hypothetical protein | Incomplete |
| NEIS0289 | hypothetical protein | Complete |
| NEIS0290 | adenylosuccinate lyase | Complete |
| NEIS0292 | conserved hypothetical protein | Complete |
| NEIS0293 | ATP-dependent DNA helicase DinG | Complete |
| NEIS0302 | 3-ketoacyl-(acyl-carrier-protein) reductase | Complete |
| NEIS0303 | GMP synthase | Incomplete |
| NEIS0304 | Lipid A export ATP-binding/permease protein (IM) | Complete |
| NEIS0305 | putative malonyl CoA-acyl carrier protein transacylase | Complete |
| NEIS0307 | 3-oxoacyl-(acyl carrier protein) synthase III | Complete |
| NEIS0310 | putative glycerol-3-phosphate acyltransferase PlsX | Complete |
| NEIS0311 | hypothetical protein | Complete |
| NEIS0312 | 50S ribosomal protein L32 | Complete |
| NEIS0313 | hypothetical protein | Complete |
| NEIS0314 | Maf-like protein | Incomplete |
| NEIS0315 | hypothetical protein | Complete |
| NEIS0316 | putative inner membrane protein translocase component YidC | Complete |
| NEIS0317 | hypothetical protein | Complete |
| NEIS0318 | ribonuclease P | Complete |
| NEIS0319 | 50S ribosomal protein L34 | Complete |
| NEIS0320 | chromosomal replication initiation protein | Incomplete |
| NEIS0321 | DNA polymerase III subunit beta | Complete |
| NEIS0323 | polyphosphate kinase | Incomplete |
| NEIS0324 | putative periplasmic protein | Complete |
| NEIS0325 | lipoprotein | Complete |
| NEIS0326 | leucyl-tRNA synthetase | Complete |
| NEIS0331 | DNA binding protein | Complete |
| NEIS0333 | preprotein translocase subunit SecG | Complete |
| NEIS0334 | triosephosphate isomerase | Complete |
| NEIS0335 | hypothetical protein | Complete |
| NEIS0336 | protein-L-isoaspartate O-methyltransferase | Incomplete |
| NEIS0337 | hypothetical protein | Complete |
| NEIS0342 | putative prolyl endopeptidase | Incomplete |
| NEIS0343 | N-acetylglutamate synthase | Incomplete |
| NEIS0344 | hypothetical protein | Incomplete |
| NEIS0345 | orotate phosphoribosyltransferase | Complete |
| NEIS0346 | hypothetical protein | Complete |
| NEIS0347 | putative acetyltransferase | Complete |
| NEIS0348 | hypothetical protein | Complete |
| NEIS0350 | fructose-1,6-bisphosphate aldolase (EC 4.1.2.13) | Complete |
| NEIS0351 | putative integrase/recombinase | Complete |
| NEIS0352 | 1-deoxy-D-xylulose-5-phosphate synthase | Incomplete |
| NEIS0353 | (dimethylallyl)adenosine tRNA methylthiotransferase | Complete |
| NEIS0354 | glutamate-1-semialdehyde aminotransferase | Complete |
| NEIS0355 | oligoribonuclease | Complete |
| NEIS0356 | ribosomal protein L11 methyltransferase | Complete |
| NEIS0357 | putative acetyl-CoA carboxylase biotin carboxylase component | Complete |
| NEIS0358 | acetyl-CoA carboxylase biotin carboxyl carrier protein subunit | Incomplete |
| NEIS0360 | S-adenosylmethionine:tRNA ribosyltransferase-isomerase | Complete |
| NEIS0363 | carbamoyl phosphate synthase large subunit (EC 6.3.5.5) | Incomplete |
| NEIS0370 | carbamoyl phosphate synthase small subunit | Complete |
| NEIS0371 | type IV pilus associated protein | Incomplete |
| NEIS0372 | hypothetical protein | Complete |
| NEIS0373 | putative periplasmic thioredoxin | Complete |
| NEIS0374 | marR family transcriptional regulator | Complete |
| NEIS0375 | putative NADH:FMN oxidoreductase | Complete |
| NEIS0376 | putative sugar-phosphate nucleotidyl transferase | Complete |
| NEIS0377 | putative integral membrane protein | Complete |
| NEIS0378 | formate--tetrahydrofolate ligase | Complete |
| NEIS0379 | conserved hypothetical protein | Complete |
| NEIS0381 | tyrosyl-tRNA synthetase | Complete |
| NEIS0382 | bifunctional riboflavin kinase/FMN adenylyltransferase | Complete |
| NEIS0383 | isoleucyl-tRNA synthetase | Incomplete |
| NEIS0384 | lipoprotein signal peptidase | Complete |
| NEIS0385 | 4-hydroxy-3-methylbut-2-enyl diphosphate reductase | Complete |
| NEIS0386 | phosphatase | Incomplete |
| NEIS0389 | DNA polymerase III, alpha subunit | Incomplete |
| NEIS0390 | hypothetical protein | Complete |
| NEIS0392 | hypothetical protein | Complete |
| NEIS0394 | hypothetical protein | Incomplete |
| NEIS0395 | valine--pyruvate transaminase | Incomplete |
| NEIS0396 | pilin glycosylation protein | Complete |
| NEIS0397 | pilin glycosylation protein | Complete |
| NEIS0402 | putative lipopolysaccharide biosynthesis translocase | Incomplete |
| NEIS0403 | putative diaminohydroxyphosphoribosylaminopyrimidine deaminase/phosphoribosylamino)uracil reductase | Complete |
| NEIS0404 | transcriptional regulator NrdR | Complete |
| NEIS0405 | hypothetical protein | Complete |
| NEIS0406 | 3-dehydroquinate synthase | Complete |
| NEIS0407 | shikimate kinase | Complete |
| NEIS0408 | type IV secretin protein | Incomplete |
| NEIS0409 | type IV biogenesis protein | Complete |
| NEIS0410 | type IV biogenesis protein | Complete |
| NEIS0411 | type IV biogenesis protein | Complete |
| NEIS0412 | type IV biogenesis protein | Complete |
| NEIS0414 | penicillin-binding protein 1 | Complete |
| NEIS0415 | ribosome biogenesis GTP-binding protein YsxC | Complete |
| NEIS0416 | putative cytochrome C | Incomplete |
| NEIS0417 | hypothetical protein | Incomplete |
| NEIS0418 | hypothetical protein | Complete |
| NEIS0419 | DNA-binding/iron metalloprotein/AP endonuclease | Incomplete |
| NEIS0421 | lipid A biosynthesis lauroyl transferase | Complete |
| NEIS0422 | S-adenosylmethionine synthetase | Complete |
| NEIS0425 | putative peptidase | Complete |
| NEIS0426 | putative oxidoreductase | Complete |
| NEIS0429 | two-component system sensor kinase | Complete |
| NEIS0430 | hypothetical protein | Complete |
| NEIS0431 | putative glutaredoxin | Complete |
| NEIS0432 | preprotein translocase subunit SecB | Complete |
| NEIS0433 | ATP-dependent DNA helicase RecG | Incomplete |
| NEIS0434 | N-acetyl-gamma-glutamyl-phosphate reductase | Incomplete |
| NEIS0462 | ribonuclease BN/unknown domain fusion protein | Complete |
| NEIS0464 | hypothetical protein | Complete |
| NEIS0465 | hypothetical protein | Complete |
| NEIS0466 | hypothetical protein | Complete |
| NEIS0467 | hypothetical protein | Complete |
| NEIS0468 | hypothetical protein | Complete |
| NEIS0469 | beta-hexosaminidase | Complete |
| NEIS0470 | putative integral membrane protein | Complete |
| NEIS0471 | putative periplasmic serine protease | Complete |
| NEIS0472 | endonuclease III | Complete |
| NEIS0473 | hypothetical protein | Complete |
| NEIS0474 | transmembrane hexose transporter | Incomplete |
| NEIS0475 | putative transmembrane transport protein | Incomplete |
| NEIS0476 | hypothetical protein | Complete |
| NEIS0477 | hypothetical protein | Complete |
| NEIS0478 | porphobilinogen deaminase | Complete |
| NEIS0479 | aromatic amino acid aminotransferase | Complete |
| NEIS0480 | hypothetical protein | Complete |
| NEIS0481 | hypothetical protein | Complete |
| NEIS0482 | putative transmembrane transport protein | Complete |
| NEIS0483 | UDP-2,3-diacylglucosamine hydrolase | Complete |
| NEIS0484 | hypothetical protein | Complete |
| NEIS0486 | alcohol dehydrogenase | Complete |
| NEIS0487 | minor pilin | Complete |
| NEIS0488 | putative periplasmic protein; macrolide-specific effux protein | Complete |
| NEIS0489 | putative ABC transporter ATP-binding protein; macrolide export ATP-binding/permease protein | Complete |
| NEIS0490 | putative thiol:disulphide interchange protein | Incomplete |
| NEIS0491 | primosome assembly protein PriA | Incomplete |
| NEIS0493 | hypothetical protein | Incomplete |
| NEIS0495 | molecular chaperone DnaK | Complete |
| NEIS0496 | hypothetical protein | Complete |
| NEIS0497 | putative transcriptional regulator | Complete |
| NEIS0498 | iron-sulfur cluster insertion protein ErpA | Complete |
| NEIS0500 | putative ubiquinone biosynthesis protein UbiB | Complete |
| NEIS0501 | serine acetyltransferase | Complete |
| NEIS0502 | heat shock protein GrpE | Incomplete |
| NEIS0503 | putative periplasmic protein | Complete |
| NEIS0504 | putative thiamine biosynthesis protein | Complete |
| NEIS0505 | Na(+)-translocating NADH-quinone reductase subunit F | Complete |
| NEIS0506 | Na(+)-translocating NADH-quinone reductase subunit E | Complete |
| NEIS0507 | Na(+)-translocating NADH-quinone reductase subunit D | Complete |
| NEIS0508 | Na(+)-translocating NADH-quinone reductase subunit C | Complete |
| NEIS0509 | Na(+)-translocating NADH-quinone reductase subunit B | Complete |
| NEIS0510 | Na(+)-translocating NADH-quinone reductase subunit A | Complete |
| NEIS0512 | hypothetical protein | Complete |
| NEIS0513 | hypothetical protein | Complete |
| NEIS0515 | AsnC family transcriptional regulator | Complete |
| NEIS0516 | glycine cleavage system aminomethyltransferase T | Complete |
| NEIS0517 | glycine cleavage system protein H | Complete |
| NEIS0518 | glutamyl-tRNA reductase | Complete |
| NEIS0521 | ABC transporter ATP-binding protein | Complete |
| NEIS0522 | lipoprotein | Complete |
| NEIS0523 | electron transfer flavoprotein-ubiquinone oxidoreductase | Complete |
| NEIS0528 | putative periplasmic binding protein | Complete |
| NEIS0529 | putative ABC-transporter membrane protein | Incomplete |
| NEIS0530 | ABC transporter ATP-binding protein | Complete |
| NEIS0531 | 50S ribosomal protein L19 | Complete |
| NEIS0532 | tRNA (guanine-N(1)-)-methyltransferase | Incomplete |
| NEIS0533 | 16S rRNA-processing protein RimM | Complete |
| NEIS0534 | 30S ribosomal protein S16 | Complete |
| NEIS0536 | putative two-component system sensor kinase | Complete |
| NEIS0537 | putative two-component system regulator | Complete |
| NEIS0539 | integral membrane protein | Complete |
| NEIS0540 | hypothetical protein | Complete |
| NEIS0541 | Maf-like protein | Complete |
| NEIS0542 | putative sec-independent protein translocase component | Complete |
| NEIS0543 | putative sec-independent protein translocase component | Complete |
| NEIS0545 | putative nucleotide-binding protein | Complete |
| NEIS0546 | phosphoribosyl-ATP pyrophosphatase | Complete |
| NEIS0547 | putative zinc-binding alcohol dehydrogenase | Complete |
| NEIS0548 | hypothetical protein | Complete |
| NEIS0549 | hypothetical protein | Complete |
| NEIS0550 | preprotein translocase subunit SecD | Complete |
| NEIS0551 | preprotein translocase subunit SecF | Complete |
| NEIS0552 | 30S ribosomal protein S15 | Complete |
| NEIS0553 | putative polyamine permease ATP-binding protein | Complete |
| NEIS0554 | polyamine permease inner membrane protein | Complete |
| NEIS0555 | putative polyamine permease inner membrane protein | Complete |
| NEIS0556 | putative oxidoreductase | Complete |
| NEIS0560 | transcription termination factor Rho | Complete |
| NEIS0561 | phosphoenolpyruvate synthase | Complete |
| NEIS0562 | hypothetical protein | Complete |
| NEIS0563 | putative phosphatase | Incomplete |
| NEIS0565 | hypothetical protein | Complete |
| NEIS0566 | outer membrane lipoprotein LolB | Complete |
| NEIS0569 | hypothetical protein | Complete |
| NEIS0570 | peptide chain release factor 3 | Complete |
| NEIS0571 | phosphoribosyl-AMP cyclohydrolase | Complete |
| NEIS0572 | imidazole glycerol phosphate synthase subunit HisF | Complete |
| NEIS0573 | 1-(5-phosphoribosyl)-5-[(5- phosphoribosylamino)methylideneamino] imidazole-4-carboxamide isomerase | Complete |
| NEIS0574 | imidazole glycerol phosphate synthase subunit HisH | Complete |
| NEIS0575 | putative phosphate acyltransferase | Complete |
| NEIS0576 | putative iron-uptake permease ATP-binding protein | Complete |
| NEIS0577 | putative iron-uptake permease inner membrane protein | Complete |
| NEIS0578 | major ferric iron binding protein | Complete |
| NEIS0579 | hypothetical protein | Complete |
| NEIS0580 | argininosuccinate lyase | Complete |
| NEIS0581 | glucose 1-phosphate uridylyltransferase | Complete |
| NEIS0582 | putative deoxyribonucleotide triphosphate pyrophosphatase | Complete |
| NEIS0583 | hypothetical protein | Complete |
| NEIS0584 | inorganic pyrophosphatase | Complete |
| NEIS0585 | dATP pyrophosphohydrolase | Complete |
| NEIS0609 | transmembrane potassium transporter | Incomplete |
| NEIS0610 | diadenosine tetraphosphatase | Complete |
| NEIS0611 | hypothetical protein | Complete |
| NEIS0612 | outer membrane protein | Complete |
| NEIS0613 | coproporphyrinogen III oxidase | Complete |
| NEIS0614 | DNA ligase | Incomplete |
| NEIS0615 | hypothetical protein | Complete |
| NEIS0617 | N-acetyl-anhydromuranmyl-L-alanine amidase | Complete |
| NEIS0618 | putative periplasmic protein | Complete |
| NEIS0619 | thymidylate kinase | Complete |
| NEIS0620 | malate oxidoreductase (EC 1.1.1.38) | Incomplete |
| NEIS0621 | tetraacyldisaccharide 4'-kinase (EC 2.7.1.130) | Complete |
| NEIS0622 | putative periplasmic protein | Incomplete |
| NEIS0623 | hypothetical protein | Complete |
| NEIS0624 | 3-deoxy-manno-octulosonate cytidylyltransferase | Complete |
| NEIS0625 | hypothetical protein | Complete |
| NEIS0628 | tryptophan synthase subunit alpha | Complete |
| NEIS0629 | acetyl-CoA carboxylase subunit beta | Complete |
| NEIS0630 | putative periplasmic protein | Complete |
| NEIS0631 | hypothetical protein | Complete |
| NEIS0632 | lipoprotein | Incomplete |
| NEIS0633 | dihydroorotase | Incomplete |
| NEIS0634 | transcription antitermination protein NusB | Complete |
| NEIS0635 | 6,7-dimethyl-8-ribityllumazine synthase | Incomplete |
| NEIS0636 | hypothetical protein | Complete |
| NEIS0637 | ribonuclease III | Incomplete |
| NEIS0638 | GTP-binding protein Era | Complete |
| NEIS0639 | N-(5'-phosphoribosyl)anthranilate isomerase | Complete |
| NEIS0640 | transcription elongation factor GreB | Complete |
| NEIS0641 | amidophosphoribosyltransferase | Complete |
| NEIS0642 | hypothetical protein | Complete |
| NEIS0643 | putative tetrapac protein | Complete |
| NEIS0644 | bifunctional folylpolyglutamate synthase/dihydrofolate synthase | Complete |
| NEIS0645 | putative transcriptional regulator | Complete |
| NEIS0646 | hypothetical protein | Complete |
| NEIS0647 | putative amino acid permease ATP-binding protein | Complete |
| NEIS0648 | dimethyladenosine transferase | Complete |
| NEIS0649 | hypothetical protein | Incomplete |
| NEIS0650 | tryptophan synthase subunit beta | Complete |
| NEIS0652 | competence protein | Incomplete |
| NEIS0653 | competence lipoprotein | Complete |
| NEIS0654 | ribosomal large subunit pseudouridine synthase D | Incomplete |
| NEIS0655 | transmembrane transport protein | Complete |
| NEIS0656 | hypothetical protein | Incomplete |
| NEIS0657 | LPS-assembly lipoprotein | Incomplete |
| NEIS0658 | DNA polymerase III subunit delta | Complete |
| NEIS0659 | hypothetical protein | Complete |
| NEIS0660 | hypothetical protein | Incomplete |
| NEIS0661 | hypothetical protein | Complete |
| NEIS0663 | RNA polymerase factor sigma-32 | Complete |
| NEIS0664 | apolipoprotein N-acyltransferase | Incomplete |
| NEIS0668 | cytochrome C | Incomplete |
| NEIS0669 | ferrochelatase | Complete |
| NEIS0671 | queuine tRNA-ribosyltransferase | Incomplete |
| NEIS0672 | threonyl-tRNA synthetase | Incomplete |
| NEIS0673 | translation initiation factor IF-3 | Incomplete |
| NEIS0674 | 50S ribosomal protein L35 | Complete |
| NEIS0675 | 50S ribosomal protein L20 | Complete |
| NEIS0676 | phenylalanyl-tRNA synthetase subunit alpha | Incomplete |
| NEIS0681 | phenylalanyl-tRNA synthetase subunit beta | Complete |
| NEIS0682 | integration host factor subunit alpha | Complete |
| NEIS0683 | hypothetical protein | Complete |
| NEIS0684 | FxsA | Complete |
| NEIS0685 | adenosylmethionine-8-amino-7-oxononanoate aminotransferase | Complete |
| NEIS0686 | dithiobiotin synthetase | Complete |
| NEIS0687 | hypothetical protein | Complete |
| NEIS0688 | 4-hydroxybenzoate octaprenyltransferase | Incomplete |
| NEIS0689 | putative regulatory protein | Complete |
| NEIS0690 | HPr kinase/phosphorylase | Incomplete |
| NEIS0691 | hypothetical protein | Complete |
| NEIS0692 | ribosomal biogenesis GTPase | Complete |
| NEIS0693 | hypothetical protein | Complete |
| NEIS0694 | putative DNA repair protein | Complete |
| NEIS0695 | hypothetical protein | Incomplete |
| NEIS0696 | hypothetical protein | Incomplete |
| NEIS0697 | ubiquinone/menaquinone biosynthesis methyltransferase | Incomplete |
| NEIS0698 | hypothetical protein | Complete |
| NEIS0699 | putative 2-amino-4-hydroxy-6-hydroxymethyldihydropteridine- pyrophosphokinase | Complete |
| NEIS0700 | putative regulator | Complete |
| NEIS0701 | hypothetical protein | Incomplete |
| NEIS0702 | putative regulator | Complete |
| NEIS0703 | putative D-alanyl-D-alanine-endopeptidase | Incomplete |
| NEIS0704 | hypothetical protein | Complete |
| NEIS0705 | putative integrase/recombinase | Complete |
| NEIS0706 | ferredoxin | Complete |
| NEIS0707 | putative thioredoxin | Complete |
| NEIS0708 | putative dTDP-4-dehydrorhamnose reductase | Complete |
| NEIS0709 | phosphoribosylaminoimidazole-succinocarboxamide synthase | Complete |
| NEIS0710 | polynucleotide phosphorylase/polyadenylase | Complete |
| NEIS0711 | hypothetical protein | Complete |
| NEIS0712 | putative lipoprotein | Complete |
| NEIS0713 | diaminopimelate epimerase | Incomplete |
| NEIS0714 | hypothetical protein | Incomplete |
| NEIS0715 | cysteine synthase | Incomplete |
| NEIS0717 | hypothetical protein | Incomplete |
| NEIS0718 | signal peptidase I | Complete |
| NEIS0719 | GTP-binding protein LepA | Complete |
| NEIS0720 | putative 5'-methylthioadenosine/S-adenosylhomocysteine nucleosidase | Complete |
| NEIS0721 | pilT_like protein | Complete |
| NEIS0722 | DNA polymerase III subunit delta' | Complete |
| NEIS0723 | type IV pilus assembly protein | Complete |
| NEIS0724 | hypothetical protein | Complete |
| NEIS0726 | hypothetical protein | Complete |
| NEIS0727 | uracil phosphoribosyltransferase | Complete |
| NEIS0728 | hypothetical protein | Complete |
| NEIS0729 | putative secreted protein | Complete |
| NEIS0730 | uroporphyrinogen-III synthase | Complete |
| NEIS0731 | hypothetical protein | Complete |
| NEIS0732 | hypothetical protein | Complete |
| NEIS0733 | uroporphyrinogen decarboxylase | Complete |
| NEIS0734 | DNA repair protein RadA | Complete |
| NEIS0735 | hypothetical protein | Complete |
| NEIS0736 | putative periplasmic protein | Complete |
| NEIS0737 | putative exodeoxyribonuclease V beta chain | Complete |
| NEIS0738 | hypothetical protein | Complete |
| NEIS0739 | putative amino acid permease substrate-binding protein | Complete |
| NEIS0741 | putative amino acid permease integral membrane protein | Complete |
| NEIS0742 | putative amino acid permease ATP-binding protein | Complete |
| NEIS0743 | Phosphoglucomutase (EC 5.4.2.2) | Complete |
| NEIS0744 | putative peptidyl-prolyl cis-trans isomerase B | Complete |
| NEIS0745 | putative transmembrane transport protein | Incomplete |
| NEIS0746 | hypothetical protein | Complete |
| NEIS0747 | peptidyl-tRNA hydrolase | Complete |
| NEIS0748 | hypothetical protein | Complete |
| NEIS0749 | hypothetical protein | Complete |
| NEIS0750 | putative ATP-dependent zinc metallopeptidase | Complete |
| NEIS0751 | putative cell division protein | Complete |
| NEIS0752 | hypothetical protein | Complete |
| NEIS0753 | delta-aminolevulinic acid dehydratase | Incomplete |
| NEIS0754 | putative carbon-sulphur lyase | Incomplete |
| NEIS0755 | putative GTP cyclohydrolase | Complete |
| NEIS0756 | putative NAD(P)H-flavin oxidoreductase | Complete |
| NEIS0757 | putative RNA-binding protein | Complete |
| NEIS0758 | inorganic polyphosphate/ATP-NAD kinase | Complete |
| NEIS0759 | hypothetical protein | Complete |
| NEIS0760 | hypothetical protein | Incomplete |
| NEIS0761 | TetR family transcriptional regulator | Complete |
| NEIS0762 | UDP-N-acetylenolpyruvoylglucosamine reductase | Complete |
| NEIS0763 | multidrug efflux protein | Complete |
| NEIS0764 | ATP phosphoribosyltransferase regulatory subunit | Complete |
| NEIS0765 | adenylosuccinate synthetase | Complete |
| NEIS0766 | heat shock protein HtpX | Complete |
| NEIS0767 | adenylate kinase | Complete |
| NEIS0768 | orotidine 5'-phosphate decarboxylase | Complete |
| NEIS0769 | D-beta-D-heptose-7-phosphate kinase | Complete |
| NEIS0773 | ADP-D-beta-D heptose epimerase | Complete |
| NEIS0774 | putative ATP-dependent protease ATP-binding protein | Complete |
| NEIS0775 | ATP-dependent Clp protease adaptor protein ClpS | Complete |
| NEIS0776 | putative transcriptional regulator | Complete |
| NEIS0777 | hypothetical protein | Complete |
| NEIS0778 | hypothetical protein | Complete |
| NEIS0779 | hypothetical protein | Complete |
| NEIS0780 | putative single-stranded-DNA-specific exonuclease | Complete |
| NEIS0781 | putative poly(A) polymerase | Complete |
| NEIS0784 | hypothetical protein | Complete |
| NEIS0788 | putative periplasmic protein | Complete |
| NEIS0789 | deoxycytidine triphosphate deaminase | Complete |
| NEIS0790 | hypothetical protein | Incomplete |
| NEIS0791 | recombination associated protein | Incomplete |
| NEIS0792 | GTP-binding protein EngA | Incomplete |
| NEIS0793 | hypothetical protein | Complete |
| NEIS0794 | histidyl-tRNA synthetase | Complete |
| NEIS0808 | hypothetical protein | Incomplete |
| NEIS0809 | hypothetical protein | Complete |
| NEIS0810 | spermidine synthase | Complete |
| NEIS0811 | 3-methyl-2-oxobutanoate hydroxymethyltransferase | Incomplete |
| NEIS0812 | pantoate--beta-alanine ligase | Complete |
| NEIS0813 | putative periplasmic protein | Complete |
| NEIS0814 | outer membrane lipoprotein LolB | Complete |
| NEIS0815 | 4-diphosphocytidyl-2-C-methyl-D-erythritol kinase | Complete |
| NEIS0816 | ribose-phosphate pyrophosphokinase | Complete |
| NEIS0817 | 50S ribosomal protein L25 | Complete |
| NEIS0818 | putative D-alanyl-D-alanine carboxypeptidase | Complete |
| NEIS0819 | threonine dehydratase | Complete |
| NEIS0823 | hypothetical protein | Complete |
| NEIS0825 | superoxide dismutase | Complete |
| NEIS0826 | replicative DNA helicase | Incomplete |
| NEIS0827 | type IV biogenesis protein | Complete |
| NEIS0828 | type IV biogenesis protein | Complete |
| NEIS0829 | type IV biogenesis protein | Complete |
| NEIS0830 | type IV biogenesis protein | Complete |
| NEIS0831 | minor pilin | Complete |
| NEIS0833 | deoxyuridine 5'-triphosphate nucleotidohydrolase | Complete |
| NEIS0834 | succinyldiaminopimelate transaminase | Complete |
| NEIS0835 | hypothetical protein | Complete |
| NEIS0897 | isocitrate dehydrogenase (NADP+) (EC 1.1.1.42) | Complete |
| NEIS0899 | alpha-2,3-sialyltransferase | Complete |
| NEIS0900 | putative C-type cytochrome | Complete |
| NEIS0901 | putative oxidoreductase | Complete |
| NEIS0902 | putative acyl-CoA hydrolase | Complete |
| NEIS0905 | proline iminopeptidase | Complete |
| NEIS0906 | putative lipoprotein | Incomplete |
| NEIS0907 | dihydrodipicolinate synthase | Complete |
| NEIS0908 | transmembrane transport protein | Complete |
| NEIS0909 | RNA methylase | Incomplete |
| NEIS0910 | hypothetical protein | Incomplete |
| NEIS0911 | putative cytosine deaminase | Complete |
| NEIS0912 | tRNA delta(2)-isopentenylpyrophosphate transferase | Incomplete |
| NEIS0913 | hypothetical protein | Complete |
| NEIS0915 | elongation factor P | Complete |
| NEIS0917 | hypothetical protein | Complete |
| NEIS0918 | homoserine O-acetyltransferase | Complete |
| NEIS0919 | 50S ribosomal protein L36 | Complete |
| NEIS0920 | 50S ribosomal protein L31 type B | Complete |
| NEIS0921 | 5,10-methylenetetrahydrofolate reductase | Complete |
| NEIS0922 | 5-methyltetrahydropteroyltriglutamate-- homocysteine S-methyltransferase | Incomplete |
| NEIS0923 | putative redoxin | Complete |
| NEIS0924 | dihydrolipoamide dehydrogenase (EC 1.8.1.4) | Complete |
| NEIS0925 | succinate dehydrogenase cytochrome B subunit (EC 1.3.99.1) | Complete |
| NEIS0926 | succinate dehydrogenase hydrophobic membrane anchor protein (EC 1.3.99.1) | Complete |
| NEIS0927 | succinate dehydrogenase flavoprotein subunit (EC 1.3.99.1) | Complete |
| NEIS0928 | succinate dehydrogenase iron-sulfur protein (EC 1.3.99.1) | Complete |
| NEIS0929 | hypothetical protein | Complete |
| NEIS0930 | citrate synthase (EC 2.3.3.1) | Complete |
| NEIS0931 | 2-oxoglutarate dehydrogenase E1 component (EC 1.2.4.2) | Complete |
| NEIS0932 | dihydrolipoamide succinyltransferase E2 component (EC 2.3.1.61) | Incomplete |
| NEIS0933 | dihydrolipoamide dehydrogenase | Complete |
| NEIS0934 | hypothetical protein | Incomplete |
| NEIS0935 | succinyl-CoA synthetase subunit beta (EC 6.2.1.5) | Complete |
| NEIS0936 | succinyl-CoA synthetase subunit alpha (EC 6.2.1.5) | Complete |
| NEIS0941 | excinuclease ABC subunit A | Incomplete |
| NEIS0942 | phosphatidylserine decarboxylase | Complete |
| NEIS0944 | putative outer-membrane receptor protein | Incomplete |
| NEIS0947 | anthranilate synthase component II | Complete |
| NEIS0948 | anthranilate phosphoribosyltransferase | Incomplete |
| NEIS0958 | pyridine nucleotide transhydrogenase | Incomplete |
| NEIS0959 | hypothetical protein | Complete |
| NEIS0960 | NAD(P) transhydrogenase subunit alpha | Incomplete |
| NEIS0961 | phosphoserine phosphatase | Complete |
| NEIS0963 | bifunctional phosphoribosylaminoimidazolecarboxamide formyltransferase/IMP cyclohydrolase | Complete |
| NEIS0980 | rubredoxin | Complete |
| NEIS0981 | putative acyl-CoA dehydrogenase | Incomplete |
| NEIS0982 | Hypothetical protein | Complete |
| NEIS0983 | hypothetical protein | Incomplete |
| NEIS0984 | D-lactate dehydrogenase | Complete |
| NEIS0985 | putative oxidoreductase | Complete |
| NEIS0986 | hypothetical protein | Incomplete |
| NEIS1007 | hypothetical protein | Incomplete |
| NEIS1009 | sulfate-binding protein | Incomplete |
| NEIS1010 | hypothetical protein | Complete |
| NEIS1011 | phosphoribosylaminoimidazole carboxylase ATPase subunit | Complete |
| NEIS1013 | anthranilate synthase component I | Complete |
| NEIS1015 | ABC transporter ATP-binding protein | Incomplete |
| NEIS1016 | DedA protein ortholog | Incomplete |
| NEIS1017 | serine hydroxymethyltransferase | Complete |
| NEIS1021 | hypothetical protein | Complete |
| NEIS1022 | fructose-1,6-bisphosphatase (EC 3.1.3.11) | Complete |
| NEIS1024 | hypothetical protein | Complete |
| NEIS1026 | putative integral membrane protein | Complete |
| NEIS1027 | dihydroneopterin aldolase | Complete |
| NEIS1028 | hypothetical protein | Complete |
| NEIS1029 | camphor resistance protein CrcB | Complete |
| NEIS1030 | putative integral membrane protein | Complete |
| NEIS1031 | putative cell-division protein | Complete |
| NEIS1032 | gamma-glutamyl phosphate reductase | Incomplete |
| NEIS1033 | gamma-glutamyl kinase | Incomplete |
| NEIS1034 | 2-isopropylmalate synthase | Complete |
| NEIS1035 | hypothetical protein | Complete |
| NEIS1036 | prolipoprotein diacylglyceryl transferase | Complete |
| NEIS1037 | hypothetical protein | Complete |
| NEIS1038 | acetylglutamate kinase | Complete |
| NEIS1039 | hypothetical protein | Complete |
| NEIS1040 | DnaA regulatory inactivator Hda | Complete |
| NEIS1063 | putative periplasmic protein | Complete |
| NEIS1065 | hypothetical protein | Complete |
| NEIS1066 | putative periplasmic protein | Complete |
| NEIS1067 | short chain dehydrogenase | Complete |
| NEIS1068 | putative oxidoreductase | Incomplete |
| NEIS1069 | putative poly-isoprenyl transferase | Incomplete |
| NEIS1070 | chaperone protein HscA | Complete |
| NEIS1072 | hypothetical protein | Complete |
| NEIS1073 | putative ferredoxin | Complete |
| NEIS1074 | hypothetical protein | Complete |
| NEIS1076 | hypothetical protein | Complete |
| NEIS1077 | hypothetical protein | Complete |
| NEIS1078 | acetyl-CoA carboxylase carboxyltransferase subunit alpha | Incomplete |
| NEIS1080 | hypothetical protein | Complete |
| NEIS1081 | RNA methyltransferase | Complete |
| NEIS1082 | lipoprotein | Complete |
| NEIS1084 | putative periplasmic protein | Complete |
| NEIS1085 | putative UDP-N-acetylmuramate: L-alanyl-gamma-D-glutamyl-meso- diaminopimelate ligase | Incomplete |
| NEIS1087 | putative biotin synthase | Incomplete |
| NEIS1090 | dihydroxy-acid dehydratase | Complete |
| NEIS1097 | hypothetical protein | Complete |
| NEIS1098 | hypothetical protein | Complete |
| NEIS1099 | putative GTP-binding protein | Complete |
| NEIS1102 | putative ribonuclease | Complete |
| NEIS1103 | inosine 5'-monophosphate dehydrogenase | Incomplete |
| NEIS1104 | putative [protein-PII] uridylyltransferase | Incomplete |
| NEIS1106 | transcriptional regulator | Complete |
| NEIS1107 | bacterioferritin B | Complete |
| NEIS1108 | bacterioferritin A | Complete |
| NEIS1110 | lipoyl synthase | Incomplete |
| NEIS1111 | lipoate-protein ligase B | Complete |
| NEIS1112 | hypothetical protein | Complete |
| NEIS1113 | putative integral membrane protein | Complete |
| NEIS1114 | putative periplasmic protein | Complete |
| NEIS1115 | hypothetical protein | Complete |
| NEIS1116 | uracil-DNA glycosylase | Incomplete |
| NEIS1125 | putative periplasmic protein | Incomplete |
| NEIS1126 | ABC transporter ATP-binding protein | Complete |
| NEIS1127 | hypothetical protein | Complete |
| NEIS1128 | homoserine dehydrogenase | Complete |
| NEIS1129 | hypothetical protein | Complete |
| NEIS1130 | putative DNA-binding protein | Complete |
| NEIS1131 | putative ATP-dependent protease | Complete |
| NEIS1132 | hypothetical protein | Incomplete |
| NEIS1133 | exodeoxyribonuclease V alpha subunit | Complete |
| NEIS1134 | putative ABC-transporter ATP-binding protein | Complete |
| NEIS1135 | putative integral membrane protein | Complete |
| NEIS1136 | hypothetical protein | Complete |
| NEIS1137 | recombination protein RecR | Complete |
| NEIS1138 | putative peptidyl-prolyl cis-trans isomerase | Complete |
| NEIS1139 | hypothetical protein | Complete |
| NEIS1140 | putative ABC-transporter ATP-binding protein | Complete |
| NEIS1142 | multifunctional tRNA nucleotidyl transferase/ 2'3'-cyclic phosphodiesterase/ 2'nucleotidase/ phosphatase | Incomplete |
| NEIS1143 | hypothetical protein | Complete |
| NEIS1144 | Holliday junction DNA helicase RuvB | Complete |
| NEIS1145 | ribulose-phosphate 3-epimerase | Incomplete |
| NEIS1146 | hypothetical protein | Incomplete |
| NEIS1147 | hypothetical protein | Incomplete |
| NEIS1148 | riboflavin synthase subunit alpha | Incomplete |
| NEIS1149 | molybdopterin-guanine dinucleotide biosynthesis protein A | Complete |
| NEIS1150 | putative two-component system sensor kinase | Complete |
| NEIS1151 | putative two-component system response regulator | Incomplete |
| NEIS1152 | phosphoribosylaminoimidazole synthetase | Complete |
| NEIS1154 | hypothetical protein | Complete |
| NEIS1155 | GTP cyclohydrolase II | Complete |
| NEIS1157 | bifunctional 3,4-dihydroxy-2-butanone 4-phosphate synthase/GTP cyclohydrolase II-like protein | Complete |
| NEIS1159 | recombination factor protein RarA | Complete |
| NEIS1164 | putative lipoprotein | Complete |
| NEIS1165 | threonine synthase | Complete |
| NEIS1166 | hypothetical protein | Complete |
| NEIS1168 | ferredoxin--NADP reductase | Complete |
| NEIS1170 | putative P-type cation-transporting ATPase | Complete |
| NEIS1171 | hypothetical protein | Complete |
| NEIS1172 | putative periplasmic protein | Incomplete |
| NEIS1173 | hypothetical protein | Complete |
| NEIS1174 | DNA repair protein RadC | Complete |
| NEIS1175 | glutamate--cysteine ligase | Complete |
| NEIS1176 | isopropylmalate isomerase large subunit | Incomplete |
| NEIS1179 | isopropylmalate isomerase small subunit | Complete |
| NEIS1182 | 3-isopropylmalate dehydrogenase | Complete |
| NEIS1183 | putative periplasmic protein | Incomplete |
| NEIS1185 | aspartate ammonia-lyase | Complete |
| NEIS1186 | putative integral membrane protein | Complete |
| NEIS1188 | putative dnaJ-family protein | Incomplete |
| NEIS1189 | hypothetical protein | Complete |
| NEIS1190 | hypothetical protein | Incomplete |
| NEIS1191 | hypothetical protein | Incomplete |
| NEIS1192 | C32 tRNA thiolase | Complete |
| NEIS1196 | hypothetical protein | Complete |
| NEIS1197 | putative cation uptake regulator | Complete |
| NEIS1198 | putative protein-tyrosine-phosphatase | Complete |
| NEIS1200 | hypothetical protein | Complete |
| NEIS1201 | hypothetical protein | Complete |
| NEIS1202 | mercuric ion binding protein | Complete |
| NEIS1203 | putative polysaccharide modification protein | Complete |
| NEIS1205 | putative periplasmic protein | Incomplete |
| NEIS1207 | long-chain-fatty-acid--CoA ligase | Complete |
| NEIS1208 | putative transmembrane transport protein | Complete |
| NEIS1210 | site-specific recombinase | Complete |
| NEIS1212 | murein hydrolase | Incomplete |
| NEIS1213 | putative oxidoreductase | Incomplete |
| NEIS1214 | transcription-repair coupling factor | Incomplete |
| NEIS1216 | aspartate alpha-decarboxylase | Complete |
| NEIS1219 | hypothetical protein | Complete |
| NEIS1220 | phosphopyruvate hydratase (EC 4.2.1.11) | Incomplete |
| NEIS1221 | hypothetical protein | Complete |
| NEIS1223 | ribonucleotide-diphosphate reductase subunit beta | Complete |
| NEIS1226 | ribonucleotide-diphosphate reductase subunit alpha | Complete |
| NEIS1229 | hypothetical protein | Complete |
| NEIS1231 | 1-acyl-sn-glycerol-3-phosphate acyltransferase | Complete |
| NEIS1232 | formamidopyrimidine-DNA glycosylase | Complete |
| NEIS1233 | hypothetical protein | Incomplete |
| NEIS1234 | putative membrane bound murein transglycosylase | Complete |
| NEIS1235 | putative ribosomal small subunit pseudouridine synthase | Complete |
| NEIS1236 | pseudo | Complete |
| NEIS1237 | cytidylate kinase | Complete |
| NEIS1238 | 30S ribosomal protein S1 | Complete |
| NEIS1239 | integration host factor subunit beta | Incomplete |
| NEIS1240 | putative transcriptional regulator | Complete |
| NEIS1241 | alcohol dehydrogenase | Complete |
| NEIS1242 | esterase D | Complete |
| NEIS1243 | putative nucleotide-binding protein | Incomplete |
| NEIS1244 | nucleoside diphosphate kinase | Complete |
| NEIS1245 | hypothetical protein | Complete |
| NEIS1246 | type IV biogenesis protein | Complete |
| NEIS1247 | 4-hydroxy-3-methylbut-2-en-1-yl diphosphate synthase | Complete |
| NEIS1248 | ATP-dependent Clp protease proteolytic subunit | Complete |
| NEIS1250 | trigger factor | Complete |
| NEIS1251 | ftsK-like cell division/stress response protein | Complete |
| NEIS1252 | uracil permease | Complete |
| NEIS1253 | hypothetical protein | Complete |
| NEIS1255 | phosphatidylserine synthase | Incomplete |
| NEIS1256 | hypothetical protein | Complete |
| NEIS1257 | 50S ribosomal protein L9 | Complete |
| NEIS1258 | 30S ribosomal protein S18 | Complete |
| NEIS1260 | 30S ribosomal protein S6 | Complete |
| NEIS1261 | thioredoxin reductase | Incomplete |
| NEIS1262 | putative cation-transporting ATPase | Complete |
| NEIS1263 | excinuclease ABC subunit C | Incomplete |
| NEIS1265 | hypothetical protein | Complete |
| NEIS1267 | tRNA (guanine-N(7)-)-methyltransferase | Complete |
| NEIS1269 | excinuclease ABC subunit B | Complete |
| NEIS1270 | putative carboxy-terminal processing protease | Incomplete |
| NEIS1271 | hypothetical protein | Complete |
| NEIS1274 | hypothetical protein | Incomplete |
| NEIS1275 | Holliday junction resolvase-like protein | Complete |
| NEIS1276 | putative hydrolase | Incomplete |
| NEIS1277 | prolyl-tRNA synthetase | Complete |
| NEIS1278 | pyruvate dehydrogenase (EC 1.2.4.1) | Complete |
| NEIS1279 | dihydrolipoamide acetyltransferase (EC 2.3.1.12) | Incomplete |
| NEIS1280 | dihydrolipoamide dehydrogenase (EC 1.8.1.4) | Incomplete |
| NEIS1281 | hypothetical protein | Complete |
| NEIS1283 | inositol monophosphate family protein | Complete |
| NEIS1284 | SpoU methylase family protein | Incomplete |
| NEIS1285 | hypothetical protein | Complete |
| NEIS1286 | SUN-family protein | Complete |
| NEIS1287 | hypothetical protein | Complete |
| NEIS1288 | putative aldehyde dehydrogenase | Incomplete |
| NEIS1289 | hypothetical protein | Complete |
| NEIS1290 | aspartyl/glutamyl-tRNA(Asp/Gln) amidotransferase subunit C | Complete |
| NEIS1291 | aspartyl/glutamyl-tRNA amidotransferase subunit A | Complete |
| NEIS1293 | aspartyl/glutamyl-tRNA amidotransferase subunit B | Complete |
| NEIS1294 | iron/sulphur-binding oxidoreductase | Complete |
| NEIS1295 | pyridoxamine 5'-phosphate oxidase | Complete |
| NEIS1296 | pseudouridine synthase | Complete |
| NEIS1297 | putative integral membrane transporter | Complete |
| NEIS1298 | exodeoxyribonuclease VII large subunit | Incomplete |
| NEIS1299 | NH(3)-dependent NAD synthetase | Incomplete |
| NEIS1300 | hypothetical protein | Complete |
| NEIS1301 | thioredoxin I | Complete |
| NEIS1302 | hypothetical protein | Complete |
| NEIS1303 | putative ATP-dependent RNA helicase | Complete |
| NEIS1304 | putative membrane lipoprotein | Incomplete |
| NEIS1305 | hypothetical protein | Complete |
| NEIS1306 | bifunctional N-succinyldiaminopimelate-aminotransferase/acetylornithine transaminase protein | Complete |
| NEIS1307 | ATP-dependent protease ATP-binding subunit ClpX | Incomplete |
| NEIS1308 | ribosome-binding factor A | Complete |
| NEIS1309 | tRNA pseudouridine synthase B | Complete |
| NEIS1312 | L-lactate dehydrogenase | Complete |
| NEIS1314 | DNA-binding protein | Complete |
| NEIS1315 | cysteine desulfurase | Complete |
| NEIS1317 | scaffold protein | Complete |
| NEIS1318 | HesB-like protein | Complete |
| NEIS1319 | chaperone protein | Complete |
| NEIS1320 | DNA gyrase subunit A | Incomplete |
| NEIS1326 | glucose-6-phosphate isomerase (EC 5.3.1.9) | Incomplete |
| NEIS1328 | putative transcriptional regulator | Incomplete |
| NEIS1329 | glucokinase | Incomplete |
| NEIS1330 | 6-phosphogluconolactonase | Complete |
| NEIS1331 | glucose-6-phosphate 1-dehydrogenase | Complete |
| NEIS1332 | phosphogluconate dehydratase | Complete |
| NEIS1333 | keto-hydroxyglutarate-aldolase/keto-deoxy- phosphogluconate aldolase | Incomplete |
| NEIS1334 | L-threonine 3-dehydrogenase (pseudogene) | Complete |
| NEIS1336 | adenine glycosylase | Incomplete |
| NEIS1347 | aminopeptidase N | Complete |
| NEIS1348 | hypothetical protein | Complete |
| NEIS1351 | lipid A biosynthesis lauroyl acyltransferase (EC 2.3.1.-) | Complete |
| NEIS1352 | Holliday junction resolvase | Complete |
| NEIS1353 | Fis family transcriptional regulator | Complete |
| NEIS1354 | hypothetical protein | Incomplete |
| NEIS1355 | putative ATP-dependent RNA helicase | Complete |
| NEIS1361 | lysyl-tRNA synthetase | Complete |
| NEIS1362 | putative integral membrane protein | Complete |
| NEIS1363 | putative aminopeptidase | Incomplete |
| NEIS1365 | transcription elongation factor GreA | Complete |
| NEIS1366 | 3-phosphoshikimate 1-carboxyvinyltransferase | Complete |
| NEIS1367 | putative lipoprotein | Complete |
| NEIS1368 | phopholipase D-family protein | Incomplete |
| NEIS1370 | putative integral membrane protein | Complete |
| NEIS1371 | hypothetical protein | Complete |
| NEIS1372 | hypothetical protein | Incomplete |
| NEIS1373 | iron-sulphur protein | Complete |
| NEIS1374 | phosphoribosylaminoimidazole carboxylase catalytic subunit | Complete |
| NEIS1375 | putative periplasmic protein | Complete |
| NEIS1376 | methyltransferase | Complete |
| NEIS1378 | DNA mismatch repair protein | Incomplete |
| NEIS1379 | DNA polymerase III subunits gamma and tau | Incomplete |
| NEIS1380 | hypothetical protein | Complete |
| NEIS1382 | recombinase A | Complete |
| NEIS1383 | 3-dehydroquinate dehydratase | Complete |
| NEIS1385 | ATP-dependent DNA helicase | Complete |
| NEIS1386 | DNA polymerase IV | Complete |
| NEIS1389 | putative ferredoxin NADP+ reductase | Complete |
| NEIS1391 | DNA polymerase III subunit | Complete |
| NEIS1392 | hypothetical protein | Incomplete |
| NEIS1393 | putative inner membrane protein | Complete |
| NEIS1394 | putative ferredoxin | Complete |
| NEIS1395 | transketolase | Complete |
| NEIS1396 | fumarate hydratase class II (EC 4.2.1.2) | Incomplete |
| NEIS1397 | hypothetical protein | Incomplete |
| NEIS1398 | single-stranded binding protein | Incomplete |
| NEIS1400 | putative integral membrane transporter | Incomplete |
| NEIS1401 | putative transglycosylase | Complete |
| NEIS1404 | hypothetical protein | Complete |
| NEIS1405 | exopolyphosphatase | Complete |
| NEIS1407 | hypothetical protein | Complete |
| NEIS1408 | tryptophanyl-tRNA synthetase | Complete |
| NEIS1409 | ClpB protein | Complete |
| NEIS1410 | aminotransferase AlaT | Complete |
| NEIS1411 | tautomerase | Complete |
| NEIS1413 | putative glutamate dehydrogenase | Complete |
| NEIS1414 | phosphoglycolate phosphatase | Complete |
| NEIS1415 | recombination regulator RecX | Complete |
| NEIS1417 | putative acyl-CoA hydrolase | Complete |
| NEIS1418 | putative membrane peptidase | Complete |
| NEIS1419 | stationary phase survival protein SurE | Complete |
| NEIS1420 | hypothetical protein | Complete |
| NEIS1422 | fimbrial assembly protein | Complete |
| NEIS1423 | succinate semialdehyde dehydrogenase | Complete |
| NEIS1425 | lipoprotein | Complete |
| NEIS1426 | putative integral membrane protein (CstA-like) | Complete |
| NEIS1429 | aspartate kinase | Incomplete |
| NEIS1430 | ribonuclease PH | Complete |
| NEIS1431 | hypothetical protein | Complete |
| NEIS1432 | hypothetical protein | Complete |
| NEIS1433 | hypothetical protein | Incomplete |
| NEIS1435 | nicotinate phosphoribosyltransferase | Incomplete |
| NEIS1436 | arginyl-tRNA synthetase | Incomplete |
| NEIS1437 | hypothetical protein | Incomplete |
| NEIS1438 | putative binding-protein-dependent transport systems inner membrane protein | Incomplete |
| NEIS1439 | putative nuclease | Incomplete |
| NEIS1440 | ribose-5-phosphate isomerase A | Incomplete |
| NEIS1441 | 2-C-methyl-D-erythritol 2,4-cyclodiphosphate synthase | Complete |
| NEIS1442 | 2-C-methyl-D-erythritol 4-phosphate cytidylyltransferase | Incomplete |
| NEIS1443 | DNA polymerase III subunit epsilon | Complete |
| NEIS1444 | putative integral membrane protein | Incomplete |
| NEIS1445 | putative inner membrane protein | Complete |
| NEIS1446 | hypothetical protein | Complete |
| NEIS1447 | acetate kinase | Incomplete |
| NEIS1448 | thiol:disulfide interchange protein precursor | Complete |
| NEIS1449 | hypothetical protein | Complete |
| NEIS1450 | putative transferase | Complete |
| NEIS1451 | putative peptidyl-prolyl isomerase | Complete |
| NEIS1453 | hypothetical protein | Complete |
| NEIS1455 | SsrA-binding protein | Complete |
| NEIS1456 | Heptosyltransferase II | Complete |
| NEIS1457 | putative methylated-DNA-protein-cysteine methyltransferase | Complete |
| NEIS1458 | hypothetical protein | Complete |
| NEIS1459 | succinyl-diaminopimelate desuccinylase | Incomplete |
| NEIS1460 | hypothetical protein | Complete |
| NEIS1461 | hypothetical protein | Complete |
| NEIS1462 | H.8 outer membrane protein | Complete |
| NEIS1464 | preprotein translocase subunit SecA | Incomplete |
| NEIS1465 | DNA primase | Complete |
| NEIS1466 | RNA polymerase sigma factor RpoD | Incomplete |
| NEIS1471 | CTP synthetase | Complete |
| NEIS1472 | long-chain-fatty-acid--CoA-ligase | Complete |
| NEIS1473 | tRNA-specific 2-thiouridylase MnmA | Complete |
| NEIS1474 | hypothetical protein | Complete |
| NEIS1475 | diacylglycerol kinase | Complete |
| NEIS1477 | glutathione synthetase | Complete |
| NEIS1478 | glutaminyl-tRNA synthetase | Complete |
| NEIS1479 | glycerol 3-phosphate regulon repressor | Complete |
| NEIS1480 | hypothetical protein | Complete |
| NEIS1481 | GntR family transcriptional regulator | Incomplete |
| NEIS1483 | hypothetical protein | Complete |
| NEIS1485 | hypothetical protein | Complete |
| NEIS1486 | phosphoribosylglycinamide transformylase | Incomplete |
| NEIS1487 | peptidylprolyl isomerase (EC:5.2.1.8) | Complete |
| NEIS1488 | DNA polymerase III chi subunit | Complete |
| NEIS1489 | aminopeptidase A | Complete |
| NEIS1490 | hypothetical protein | Complete |
| NEIS1491 | hypothetical protein | Complete |
| NEIS1492 | aconitate hydratase 2 (EC 4.2.1.3) | Complete |
| NEIS1493 | ornithine carbamoyltransferase | Complete |
| NEIS1494 | ketol-acid reductoisomerase | Complete |
| NEIS1495 | hypothetical protein | Complete |
| NEIS1496 | acetolactate synthase 3 regulatory subunit | Complete |
| NEIS1497 | acetolactate synthase isozyme III large subunit | Complete |
| NEIS1498 | putative lipoprotein | Complete |
| NEIS1499 | ATP phosphoribosyltransferase catalytic subunit | Complete |
| NEIS1501 | histidinol dehydrogenase | Complete |
| NEIS1502 | histidinol-phosphate aminotransferase | Complete |
| NEIS1503 | imidazoleglycerol-phosphate dehydratase | Incomplete |
| NEIS1504 | hypothetical protein | Complete |
| NEIS1505 | transcriptional regulator | Complete |
| NEIS1508 | putative periplasmic protease | Complete |
| NEIS1509 | CDP-diacylglycerol--glycerol-3-phosphate 3-phosphatidyltransferase | Complete |
| NEIS1511 | hypothetical protein | Complete |
| NEIS1512 | hypothetical protein | Complete |
| NEIS1513 | AraC family transcription regulator | Complete |
| NEIS1514 | hypothetical protein | Complete |
| NEIS1515 | hypothetical protein | Complete |
| NEIS1518 | alanyl-tRNA synthetase | Complete |
| NEIS1524 | phosphoglycerate mutase (EC 5.4.2.1) | Complete |
| NEIS1525 | DNA topoisomerase IV subunit A | Complete |
| NEIS1526 | two component sensor kinase | Incomplete |
| NEIS1528 | hypothetical protein | Complete |
| NEIS1530 | O-succinylhomoserine sulfhydrolase | Incomplete |
| NEIS1531 | hypothetical protein | Complete |
| NEIS1532 | hypothetical protein | Complete |
| NEIS1533 | histidine-binding periplasmic protein | Complete |
| NEIS1534 | fumarate hydratase class I (EC 4.2.1.2) | Incomplete |
| NEIS1535 | potassium transporter peripheral membrane component | Incomplete |
| NEIS1538 | phosphomethylpyrimidine kinase | Complete |
| NEIS1544 | ribonuclease H | Complete |
| NEIS1545 | hypothetical protein | Complete |
| NEIS1546 | hypothetical protein | Complete |
| NEIS1548 | nitric oxide reductase | Incomplete |
| NEIS1550 | hypothetical protein | Complete |
| NEIS1552 | hypothetical protein | Complete |
| NEIS1554 | phosphoserine aminotransferase | Complete |
| NEIS1555 | hypothetical protein | Complete |
| NEIS1556 | transcription elongation factor NusA | Complete |
| NEIS1557 | translation initiation factor IF-2 | Incomplete |
| NEIS1558 | putative integral membrane protein | Incomplete |
| NEIS1559 | putative integral membrane protein | Complete |
| NEIS1560 | hemolysin | Incomplete |
| NEIS1562 | putative sodium:alanine symporter | Complete |
| NEIS1563 | hypothetical protein | Complete |
| NEIS1564 | putative disulphide bond formation protein | Complete |
| NEIS1566 | transcription regulator AsnC | Complete |
| NEIS1567 | alanine racemase | Complete |
| NEIS1568 | hypothetical protein | Incomplete |
| NEIS1569 | hypothetical protein | Complete |
| NEIS1570 | putative periplasmic protein | Complete |
| NEIS1571 | N5-glutamine S-adenosyl-L-methionine-dependent methyltransferase | Complete |
| NEIS1576 | hypothetical protein | Complete |
| NEIS1577 | guanosine-3',5'-bis(diphosphate) 3'-pyrophophohydrolase | Incomplete |
| NEIS1578 | DNA-directed RNA polymerase omega chain | Complete |
| NEIS1579 | guanylate kinase | Complete |
| NEIS1580 | adenine phosphoribosyltransferase | Complete |
| NEIS1581 | hypothetical protein | Complete |
| NEIS1582 | protease | Incomplete |
| NEIS1583 | hypothetical protein | Incomplete |
| NEIS1587 | haem utilisation protein | Incomplete |
| NEIS1588 | Putative paraquat-inducible protein A | Complete |
| NEIS1589 | Putative paraquat-inducible protein B | Complete |
| NEIS1590 | putative lipoprotein | Complete |
| NEIS1591 | DNA-3-methyladenine glycosylase I | Complete |
| NEIS1592 | putative lipase | Complete |
| NEIS1594 | glycine dehydrogenase | Complete |
| NEIS1595 | putative cytochrome | Complete |
| NEIS1596 | aromatic amino acid aminotransferase | Complete |
| NEIS1597 | tRNA (uracil-5-)-methyltransferase | Complete |
| NEIS1598 | chorismate synthase | Complete |
| NEIS1599 | hypothetical protein | Complete |
| NEIS1600 | DNA topoisomerase IV subunit B | Complete |
| NEIS1601 | dinucleoside polyphosphate hydrolase | Complete |
| NEIS1602 | seryl-tRNA synthetase | Complete |
| NEIS1603 | D-lactate dehydrogenase | Complete |
| NEIS1604 | peptide chain release factor 1 | Complete |
| NEIS1605 | hypothetical protein | Complete |
| NEIS1606 | L-asparaginase | Complete |
| NEIS1607 | DedA family integral membrane protein | Complete |
| NEIS1608 | phosphoglucosamine mutase | Incomplete |
| NEIS1609 | dihydropteroate synthase | Complete |
| NEIS1610 | hypothetical protein | Incomplete |
| NEIS1611 | 3-octaprenyl-4-hydroxybenzoate carboxy-lyase | Complete |
| NEIS1613 | hypothetical protein | Complete |
| NEIS1614 | hypothetical protein | Complete |
| NEIS1617 | 3-oxoacyl-(acyl carrier protein) synthase II | Complete |
| NEIS1622 | putative integral membrane ion transporter | Complete |
| NEIS1624 | thymidylate synthase | Complete |
| NEIS1625 | glutamate dehydrogenase | Complete |
| NEIS1629 | GntR family transcriptional regulator | Complete |
| NEIS1632 | putative outer membrane lipoprotein | Complete |
| NEIS1633 | drug efflux protein | Complete |
| NEIS1634 | membrane fusion protein | Complete |
| NEIS1635 | transcriptional regulator | Incomplete |
| NEIS1638 | exodeoxyribonuclease V | Complete |
| NEIS1639 | putative integral membrane protein | Incomplete |
| NEIS1640 | Cytochrome c oxidase subunit III / Cbb3-type cytochrome c oxidase subunit | Incomplete |
| NEIS1643 | cbb3-type cytochrome c oxidase subunit II | Complete |
| NEIS1645 | cbb3-type cytochrome c oxidase subunit I | Complete |
| NEIS1646 | glycyl aminopeptidase | Incomplete |
| NEIS1647 | hypothetical protein | Complete |
| NEIS1648 | biopolymer transport protein | Complete |
| NEIS1649 | biopolymer transport protein | Complete |
| NEIS1650 | TonB protein | Complete |
| NEIS1651 | hypothetical protein | Complete |
| NEIS1654 | glutaredoxin 2 | Complete |
| NEIS1655 | GTP pyrophosphokinase | Complete |
| NEIS1669 | hypothetical protein | Complete |
| NEIS1670 | hypothetical protein | Incomplete |
| NEIS1671 | hypothetical protein | Complete |
| NEIS1673 | hypothetical protein | Complete |
| NEIS1677 | hypothetical protein | Complete |
| NEIS1679 | putative integral membrane transport protein | Complete |
| NEIS1683 | hypothetical protein | Complete |
| NEIS1684 | aspartyl-tRNA synthetase | Complete |
| NEIS1686 | putative integral membrane protein | Complete |
| NEIS1687 | outer membrane phospholipase A precursor (ec 3.1.1.32) | Incomplete |
| NEIS1688 | 30S ribosomal protein S20 | Complete |
| NEIS1689 | putative polyamine permease substrate-binding protein | Complete |
| NEIS1692 | glutamate racemase | Complete |
| NEIS1693 | hypothetical protein | Complete |
| NEIS1694 | N-acetylmuramoyl-L-alanine amidase | Complete |
| NEIS1697 | hypothetical protein | Complete |
| NEIS1698 | putative secreted protein | Complete |
| NEIS1699 | MutT-related protein | Complete |
| NEIS1700 | 4'-phosphopantetheinyl transferase | Complete |
| NEIS1703 | pyridoxine 5'-phosphate synthase | Complete |
| NEIS1704 | DNA repair protein (recombination protein o) | Complete |
| NEIS1705 | chorismate mutase | Complete |
| NEIS1706 | integral membrane efflux protein | Complete |
| NEIS1708 | hypothetical protein | Complete |
| NEIS1720 | hypothetical protein | Complete |
| NEIS1721 | oxidoreductase | Complete |
| NEIS1722 | hypothetical protein | Complete |
| NEIS1724 | hypothetical protein | Complete |
| NEIS1725 | hypothetical protein | Complete |
| NEIS1726 | hypothetical protein | Complete |
| NEIS1735 | putative permease | Incomplete |
| NEIS1737 | cell division protein FtsZ | Complete |
| NEIS1738 | cell division protein | Complete |
| NEIS1739 | cell division protein | Complete |
| NEIS1740 | D-alanine--D-alanine ligase | Complete |
| NEIS1741 | UDP-N-acetylmuramate--L-alanine ligase | Complete |
| NEIS1742 | undecaprenyldiphospho-muramoylpentapeptide beta-N- acetylglucosaminyltransferase | Complete |
| NEIS1743 | cell division protein | Incomplete |
| NEIS1745 | UDP-N-acetylmuramoyl-L-alanyl-D-glutamate synthetase | Complete |
| NEIS1747 | phospho-N-acetylmuramoyl-pentapeptide- transferase | Complete |
| NEIS1748 | putative periplasmic protein | Complete |
| NEIS1749 | UDP-MurNAc-pentapeptide synthetase | Complete |
| NEIS1751 | UDP-N-acetylmuramoylalanyl-D-glutamate--2, 6-diaminopimelate ligase | Incomplete |
| NEIS1753 | penicillin-binding protein 2 | Incomplete |
| NEIS1754 | putative small periplasmic protein | Complete |
| NEIS1755 | S-adenosyl-methyltransferase MraW | Complete |
| NEIS1756 | cell division protein MraZ | Complete |
| NEIS1757 | hypothetical protein | Incomplete |
| NEIS1759 | undecaprenyl pyrophosphate phosphatase | Complete |
| NEIS1760 | putative thiol:disulphide interchange protein DsbA3; oxidoreductase | Complete |
| NEIS1762 | hypothetical protein | Complete |
| NEIS1763 | putative chelatase | Complete |
| NEIS1764 | hypothetical protein | Complete |
| NEIS1765 | sodium/proline symporter | Complete |
| NEIS1766 | bifunctional proline dehydrogenase/pyrroline-5-carboxylate dehydrogenase | Incomplete |
| NEIS1768 | exodeoxyribonuclease III | Complete |
| NEIS1769 | ArsR family transcriptional regulator | Complete |
| NEIS1770 | nicotinate-nucleotide pyrophosphorylase | Complete |
| NEIS1771 | putative cytoplasmic membrane protein | Complete |
| NEIS1772 | quinolinate synthetase | Incomplete |
| NEIS1773 | L-aspartate oxidase | Incomplete |
| NEIS1776 | beta-phosphoglucomutase | Complete |
| NEIS1777 | maltose phosphorylase | Incomplete |
| NEIS1779 | integral membrane transport protein | Incomplete |
| NEIS1780 | putative ABC transporter ATP-binding protein | Complete |
| NEIS1781 | phosphatidylglycerophosphatase A | Complete |
| NEIS1782 | thiamine monophosphate kinase | Incomplete |
| NEIS1783 | outer membrane protein class 4 | Complete |
| NEIS1784 | transcriptional regulator CysB-like protein | Complete |
| NEIS1785 | putative anaerobic transcriptional regulatory protein | Complete |
| NEIS1786 | coproporphyrinogen III oxidase | Complete |
| NEIS1787 | putative phosphate permease | Complete |
| NEIS1788 | anhydro-N-acetylmuramic acid kinase | Incomplete |
| NEIS1804 | hypothetical periplasmic protein | Complete |
| NEIS1807 | hypothetical protein | Complete |
| NEIS1808 | putative integral membrane signal transducer protein | Complete |
| NEIS1809 | glutamine synthetase | Complete |
| NEIS1810 | shikimate dehydrogenase | Complete |
| NEIS1811 | monofunctional biosynthetic peptidoglycan transglycosylase | Complete |
| NEIS1812 | lipopolysaccharide ABC transporter | Complete |
| NEIS1813 | LPS-assembly protein LptD | Complete |
| NEIS1814 | lipopolysaccharide export system protein (OM) | Complete |
| NEIS1815 | 3-deoxy-D-manno-octulosonate 8-phosphate phosphatase, KDO 8-P phosphatase (EC 3.1.3.45) | Complete |
| NEIS1816 | arabinose-5-phosphate isomerase (EC 5.3.1.13) | Complete |
| NEIS1818 | transaldolase | Complete |
| NEIS1820 | glutamyl-Q tRNA(Asp) synthetase | Incomplete |
| NEIS1822 | tRNA-dihydrouridine synthase A | Complete |
| NEIS1823 | D-tyrosyl-tRNA(Tyr) deacylase | Complete |
| NEIS1824 | hypothetical protein | Complete |
| NEIS1825 | hypothetical protein | Complete |
| NEIS1826 | hypothetical protein | Complete |
| NEIS1827 | YciI-like protein | Complete |
| NEIS1828 | intracellular septation protein A | Complete |
| NEIS1830 | lactoylglutathione lyase | Incomplete |
| NEIS1831 | tetratricopeptide repeat protein | Complete |
| NEIS1832 | hypothetical protein | Complete |
| NEIS1833 | branched-chain amino acid aminotransferase | Complete |
| NEIS1834 | enoyl-(acyl carrier protein) reductase | Complete |
| NEIS1835 | 2,3,4,5-tetrahydropyridine-2,6-carboxylate N-succinyltransferase | Complete |
| NEIS1837 | glucose-6-phosphate isomerase 2 (EC 5.3.1.9) | Complete |
| NEIS1838 | type IV biogenesis protein | Complete |
| NEIS1839 | type IV prepilin like protein leader peptide processing enzyme | Complete |
| NEIS1841 | hypothetical protein | Complete |
| NEIS1842 | hypothetical protein | Complete |
| NEIS1844 | type IV biogenesis protein | Complete |
| NEIS1845 | hypothetical protein | Incomplete |
| NEIS1846 | octaprenyl-diphosphate synthase | Complete |
| NEIS1847 | 50S ribosomal protein L21 | Complete |
| NEIS1848 | 50S ribosomal protein L27 | Complete |
| NEIS1849 | hypothetical protein | Complete |
| NEIS1850 | 50S ribosomal protein L33 | Complete |
| NEIS1851 | 50S ribosomal protein L28 | Complete |
| NEIS1852 | multidrug resistance translocase; fatty acid efflux system protein | Incomplete |
| NEIS1853 | multidrug resistance translocase; fatty acid efflux system protein | Incomplete |
| NEIS1854 | 7-cyano-7-deazaguanine reductase | Complete |
| NEIS1855 | hypothetical protein | Complete |
| NEIS1856 | hypothetical protein | Incomplete |
| NEIS1857 | hypothetical protein | Complete |
| NEIS1858 | hypothetical protein | Incomplete |
| NEIS1870 | hypothetical protein | Complete |
| NEIS1871 | hypothetical protein | Complete |
| NEIS1872 | hypothetical protein | Complete |
| NEIS1873 | dihydrofolate reductase | Complete |
| NEIS1882 | hypothetical protein | Complete |
| NEIS1883 | signal recognition particle protein | Complete |
| NEIS1889 | hypothetical protein | Complete |
| NEIS1890 | hypothetical protein | Complete |
| NEIS1891 | LysR family transcriptional regulator | Complete |
| NEIS1892 | deoxyribodopyrimidine photolyase | Incomplete |
| NEIS1896 | hypothetical protein | Complete |
| NEIS1898 | hypothetical protein | Complete |
| NEIS1899 | 16S ribosomal RNA methyltransferase RsmE | Complete |
| NEIS1900 | lacto-N-neotetraose biosynthesis glycosyl transferase | Incomplete |
| NEIS1902 | lacto-N-neotetraose biosynthesis glycosyl transferase | Incomplete |
| NEIS1903 | glycyl-tRNA synthetase subunit beta | Complete |
| NEIS1904 | glycyl-tRNA synthetase subunit alpha | Complete |
| NEIS1905 | F0F1 ATP synthase subunit epsilon | Incomplete |
| NEIS1906 | F0F1 ATP synthase subunit beta | Complete |
| NEIS1907 | F0F1 ATP synthase subunit gamma | Incomplete |
| NEIS1908 | F0F1 ATP synthase subunit alpha | Incomplete |
| NEIS1909 | F0F1 ATP synthase subunit delta | Complete |
| NEIS1910 | F0F1 ATP synthase subunit B | Complete |
| NEIS1911 | F0F1 ATP synthase subunit C | Complete |
| NEIS1912 | F0F1 ATP synthase subunit A | Incomplete |
| NEIS1913 | putative ATP synthase I | Complete |
| NEIS1915 | putative chromosome segregation proteins | Complete |
| NEIS1916 | aromatic acid decarboxylase | Complete |
| NEIS1917 | putative lipoprotein | Incomplete |
| NEIS1918 | inner membrane transport protein | Incomplete |
| NEIS1919 | putative ABC transport ATP-binding subunit | Complete |
| NEIS1920 | putative transglycosylase | Incomplete |
| NEIS1921 | 30S ribosomal protein S21 | Complete |
| NEIS1922 | hypothetical protein | Complete |
| NEIS1924 | ClpXP protease specificity-enhancing factor | Complete |
| NEIS1925 | pilE expression regulator/putative sspA-like protein | Complete |
| NEIS1926 | putative inner membrane protein | Incomplete |
| NEIS1928 | 50S ribosomal protein L31 | Complete |
| NEIS1929 | acetyltransferase | Complete |
| NEIS1930 | putative periplasmic protein | Complete |
| NEIS1931 | hypothetical protein | Incomplete |
| NEIS1932 | hypothetical protein | Incomplete |
| NEIS1933 | periplasmic/outer membrane protein | Incomplete |
| NEIS1934 | hypothetical protein | Incomplete |
| NEIS1935 | putative periplasmic transport protein | Incomplete |
| NEIS1936 | putative outer membrane transport protein | Complete |
| NEIS1937 | putative ABC transport inner membrane subunit | Complete |
| NEIS1938 | putative ABC transport ATP-binding protein | Incomplete |
| NEIS1942 | aldehyde dehydrogenase | Complete |
| NEIS1944 | putative para-aminobenzoate synthase component I | Complete |
| NEIS1945 | hypothetical protein | Incomplete |
| NEIS1948 | chaperonin GroEL | Incomplete |
| NEIS1949 | co-chaperonin GroES | Complete |
| NEIS1951 | putative sodium-dependent inner membrane transport protein | Complete |
| NEIS1952 | diaminopimelate decarboxylase | Complete |
| NEIS1953 | putative lipoprotein | Complete |
| NEIS1954 | frataxin-like protein | Complete |
| NEIS1955 | hypothetical protein | Complete |
| NEIS1956 | S-ribosylhomocysteinase | Complete |
| NEIS1957 | DNA polymerase I | Incomplete |
| NEIS1962 | tRNA modification GTPase TrmE | Complete |
| NEIS1964 | putative membrane transport solute-binding protein | Complete |
| NEIS1965 | putative inner membrane transport protein | Incomplete |
| NEIS1966 | putative inner membrane transport protein | Incomplete |
| NEIS1967 | hypothetical protein | Complete |
| NEIS1968 | putative ABC transporter ATP-binding subunit | Complete |
| NEIS1971 | nitrogen regulatory protein P-II 1 | Complete |
| NEIS1972 | phosphoribosylformylglycinamidine synthase | Complete |
| NEIS1973 | hydroxyacylglutathione hydrolase | Complete |
| NEIS1976 | putative magnesium transporter | Complete |
| NEIS1977 | Hsp33-like chaperonin | Complete |
| NEIS1978 | hypothetical protein | Incomplete |
| NEIS1979 | hypothetical protein | Complete |
| NEIS1980 | hypothetical protein | Complete |
| NEIS1981 | hypothetical protein | Incomplete |
| NEIS1982 | bifunctional ornithine acetyltransferase/N-acetylglutamate synthase protein | Incomplete |
| NEIS1983 | hypothetical protein | Complete |
| NEIS1984 | ATP-dependent DNA helicase | Complete |
| NEIS1987 | putative DNA helicase | Incomplete |
| NEIS1995 | minor pilin | Complete |
| NEIS1997 | psedouridine synthase | Complete |
| NEIS1998 | phosphopantetheine adenylyltransferase | Complete |
| NEIS1999 | putative integral membrane protein | Complete |
| NEIS2000 | hypothetical protein | Complete |
| NEIS2001 | rRNA large subunit methyltransferase | Complete |
| NEIS2002 | hypothetical protein | Complete |
| NEIS2003 | Probable nicotinate-nucleotide adenylyltransferase EC=2.7.7.18 | Complete |
| NEIS2004 | hypothetical protein | Complete |
| NEIS2008 | homoserine kinase | Incomplete |
| NEIS2009 | 3-demethylubiquinone-9 3-methyltransferase | Complete |
| NEIS2010 | amino-acid transport protein | Complete |
| NEIS2014 | D-alpha,beta,D-Heptose 1,7 bisphosphate phosphatase | Complete |
| NEIS2015 | 1-acyl-SN-glycerol-3-phosphate acyltransferase | Complete |
| NEIS2016 | hypothetical protein | Complete |
| NEIS2017 | tRNA pseudouridine synthase A | Complete |
| NEIS2020 | PorB, porin, major outer membrane protein | Complete |
| NEIS2021 | thiamine biosynthesis protein ThiC | Complete |
| NEIS2022 | hypothetical protein | Complete |
| NEIS2023 | ABC-transport system ATP-binding protein | Complete |
| NEIS2024 | phosphoenolpyruvate-protein phosphotransferase | Complete |
| NEIS2025 | sugar transport PTS system phosphocarrier protein HPr | Complete |
| NEIS2026 | sugar transport PTS system IIA component | Incomplete |
| NEIS2027 | hypoxanthine-guanine phosphoribosyltransferase | Complete |
| NEIS2028 | DNA ligase | Complete |
| NEIS2029 | putative hydrolase | Complete |
| NEIS2030 | putative periplasmic protein | Incomplete |
| NEIS2032 | cytochrome C1 precursor | Complete |
| NEIS2033 | cytochrome B | Complete |
| NEIS2034 | ubiquinol-cytochrome c reductase iron-sulfur subunit | Complete |
| NEIS2035 | hypothetical protein | Complete |
| NEIS2036 | putative transcriptional activator protein METR | Complete |
| NEIS2037 | 30S ribosomal protein S9 | Complete |
| NEIS2038 | 50S ribosomal protein L13 | Complete |
| NEIS2039 | hypothetical protein | Complete |
| NEIS2040 | hypothetical protein | Complete |
| NEIS2041 | NAD(P)H-dependent glycerol-3-phosphate dehydrogenase | Complete |
| NEIS2042 | phosphoenolpyruvate carboxylase | Incomplete |
| NEIS2044 | hypothetical protein | Complete |
| NEIS2045 | putative integral membrane protein | Complete |
| NEIS2046 | hemk protein | Complete |
| NEIS2047 | TLDD protein | Complete |
| NEIS2049 | putative oxidoreductase | Complete |
| NEIS2050 | thiamin-phosphate pyrophosphorylase | Complete |
| NEIS2051 | hypothetical protein | Complete |
| NEIS2052 | thiazole synthase | Complete |
| NEIS2053 | putative periplasmic protein | Complete |
| NEIS2054 | bifunctional biotin--[acetyl-CoA-carboxylase] ligase/pantothenate kinase | Incomplete |
| NEIS2055 | D-beta-D-heptose-1-phosphate adenylyltransferase | Complete |
| NEIS2056 | bifunctional 5,10-methylene-tetrahydrofolate dehydrogenase/ 5,10-methylene-tetrahydrofolate cyclohydrolase | Complete |
| NEIS2057 | putative integral membrane protein | Complete |
| NEIS2058 | aspartate-semialdehyde dehydrogenase | Complete |
| NEIS2059 | hypothetical protein | Complete |
| NEIS2060 | hypothetical protein | Complete |
| NEIS2061 | putative nuclease | Complete |
| NEIS2062 | cysteinyl-tRNA synthetase | Incomplete |
| NEIS2065 | GTPase ObgE | Incomplete |
| NEIS2069 | hypothetical protein | Complete |
| NEIS2070 | sedoheptulose-7-phosphate isomerase | Complete |
| NEIS2071 | putative lipoprotein | Complete |
| NEIS2072 | putative periplasmic protein | Incomplete |
| NEIS2073 | methionine aminopeptidase | Complete |
| NEIS2074 | hypothetical protein | Incomplete |
| NEIS2075 | putative adhesin complex protein | Complete |
| NEIS2076 | malate:quinone oxidoreductase | Incomplete |
| NEIS2077 | hypothetical protein | Complete |
| NEIS2078 | hypothetical protein | Complete |
| NEIS2079 | putative integral membrane protein | Complete |
| NEIS2080 | 30S ribosomal protein S2 | Complete |
| NEIS2081 | elongation factor Ts | Complete |
| NEIS2082 | uridine monophosphate kinase | Complete |
| NEIS2103 | putative protease | Complete |
| NEIS2104 | hypothetical protein | Complete |
| NEIS2106 | argininosuccinate synthase | Complete |
| NEIS2110 | serine/threonine transporter SstT | Incomplete |
| NEIS2112 | putative outer membrane protein | Incomplete |
| NEIS2113 | putative periplasmic protein | Incomplete |
| NEIS2114 | peptide transporter | Complete |
| NEIS2116 | peptide chain release factor 2 | Incomplete |
| NEIS2118 | lipoprotein | Incomplete |
| NEIS2119 | putative integral membrane protein | Complete |
| NEIS2120 | putative periplasmic protein | Complete |
| NEIS2121 | hypothetical protein | Complete |
| NEIS2122 | hypothetical protein | Complete |
| NEIS2123 | RNA polymerase sigma factor | Complete |
| NEIS2124 | lipoprotein | Incomplete |
| NEIS2128 | hypothetical protein | Complete |
| NEIS2129 | phosphoribosylamine--glycine ligase | Complete |
| NEIS2130 | hypothetical protein | Complete |
| NEIS2131 | hypothetical protein | Complete |
| NEIS2132 | electron transfer flavoprotein alpha-subunit | Complete |
| NEIS2133 | electron transfer flavoprotein beta-subunit | Complete |
| NEIS2134 | Heptosyl transferase I | Incomplete |
| NEIS2135 | putative nicotinamidase | Complete |
| NEIS2136 | hypothetical protein | Complete |
| NEIS2137 | glyceraldehyde 3-phosphate dehydrogenase C (EC 1.2.1.12) | Complete |
| NEIS2138 | DNA mismatch repair protein MutS | Complete |
| NEIS2140 | putative periplasmic protein | Complete |
| NEIS2141 | glutamyl-tRNA synthetase | Complete |
| NEIS2142 | putative periplasmic protein | Complete |
| NEIS2143 | putative oxidoreductase | Complete |
| NEIS2144 | putative periplasmic thiredoxin | Complete |
| NEIS2145 | ABC transporter ATP-binding protein | Complete |
| NEIS2146 | putative ABC transporter integral membrane protein | Complete |
| NEIS2147 | hypothetical protein | Complete |
| NEIS2148 | phosphoglycerate kinase (EC 2.7.2.3) | Incomplete |
| NEIS2149 | UDP-N-acetylglucosamine 1-carboxyvinyltransferase | Complete |
| NEIS2150 | putative transmembrane transport protein | Complete |
| NEIS2151 | putative integral membrane protein | Complete |
| NEIS2152 | 3-deoxy-D-manno-octulosonic-acid transferase | Complete |
| NEIS2153 | 6-phosphogluconate dehydrogenase | Complete |
| NEIS2169 | cseE / 3-deoxy-D-manno-octulosonic acid 8-phosphate synthase | Complete |
| NEIS2375 | sec-independent protein translocase TatA/E component | Complete |
| NEIS2445 | hypothetical protein | Incomplete |
| NEIS2447 | hypothetical protein | Complete |
| NEIS2448 | hypothetical protein | Complete |
| NEIS2515 | hypothetical protein | Complete |
| NEIS2522 | hypothetical protein; DUF2788 | Complete |
| NEIS2526 | conserved hypothetical protein | Complete |
| NEIS2530 | conserved hypothetical protein | Complete |
| NEIS2553 | conserved hypothetical protein | Complete |
| NEIS2558 | conserved hypothetical integral membrane protein | Complete |
| NEIS2779 | copper-containing nitrite reductase | Complete |
| NEIS2852 | Cbb3-type cytochrome oxidase component FixQ | Complete |
| NEIS2903 | hypothetical protein | Complete |
| NEIS0007 | methionyl-tRNA synthetase | Incomplete |
| NEIS0033 | type IV pilus associated protein | Incomplete |
| NEIS0041 | DNA transport competence protein | Incomplete |
| NEIS0046 | glucose-1-phosphate thymidylyltransferase | Complete |
| NEIS0047 | dTDP-D-glucose 4,6-dehydratase | Incomplete |
| NEIS0062 | truncated galE | Complete |
| NEIS0065 | dTDP-4-keto-6-deoxy-D-glucose-3,6-epimerase | Incomplete |
| NEIS0096 | lipoprotein | Complete |
| NEIS0117 | secE | Complete |
| NEIS0139 | 50S ribosomal protein L16 | Complete |
| NEIS0142 | 50S ribosomal protein L14 | Complete |
| NEIS0148 | 50S ribosomal protein L18 | Complete |
| NEIS0151 | 50S ribosomal protein L15 | Complete |
| NEIS0153 | infA; translation initiation factor IF-1 | Complete |
| NEIS0154 | 50S ribosomal protein L36 | Complete |
| NEIS0156 | 30S ribosomal protein S11 | Complete |
| NEIS0171 | UDP-3-O-[3-hydroxymyristoyl] glucosamine N-acyltransferase (EC 2.3.1.-) | Incomplete |
| NEIS0194 | hypothetical protein | Complete |
| NEIS0212 | RNA polymerase sigma factor | Complete |
| NEIS0213 | pilin glycosyltransferase | Incomplete |
| NEIS0214 | inner membrane transport protein | Complete |
| NEIS0221 | MafI immunity protein | Complete |
| NEIS0291 | LOS O-acetyltransferase | Complete |
| NEIS0294 | hypothetical protein | Complete |
| NEIS0308 | hypothetical protein | Incomplete |
| NEIS0309 | hypothetical protein | Complete |
| NEIS0332 | hypothetical protein | Complete |
| NEIS0388 | esterase | Incomplete |
| NEIS0428 | two-component system response regulator | Complete |
| NEIS0499 | hypothetical protein | Complete |
| NEIS0519 | regulatory protein | Complete |
| NEIS0520 | hypothetical protein | Complete |
| NEIS0535 | hypothetical protein | Incomplete |
| NEIS0567 | putative polyamine permease substrate-binding protein | Complete |
| NEIS0596 | MafA2 adhesin | Complete |
| NEIS0599 | alternative toxic C-terminal extremity | Incomplete |
| NEIS0601 | putative mafS2 cassette | Complete |
| NEIS0626 | hypothetical protein | Complete |
| NEIS0665 | hypothetical protein | Incomplete |
| NEIS0667 | hypothetical protein; | Incomplete |
| NEIS0832 | hypothetical protein | Incomplete |
| NEIS0916 | lipoprotein | Complete |
| NEIS1020 | hypothetical protein | Complete |
| NEIS1041 | ABC transporter ATP-binding protein | Complete |
| NEIS1062 | ABC transporter ATP-binding protein | Complete |
| NEIS1071 | hypothetical protein | Complete |
| NEIS1088 | hypothetical protein | Incomplete |
| NEIS1089 | hypothetical protein | Complete |
| NEIS1204 | putative periplasmic protein | Incomplete |
| NEIS1218 | 2-dehydro-3-deoxyphosphooctonate aldolase (EC 2.5.1.55) | Complete |
| NEIS1222 | iron-sulfur binding protein | Complete |
| NEIS1259 | primosomal replication protein | Complete |
| NEIS1273 | lipoprotein | Complete |
| NEIS1325 | hypothetical protein | Incomplete |
| NEIS1338 | pseudo | Complete |
| NEIS1424 | hypothetical protein | Incomplete |
| NEIS1452 | hypothetical protein | Complete |
| NEIS1507 | hypothetical protein | Complete |
| NEIS1516 | putative polyamine permease substrate-binding protein | Complete |
| NEIS1527 | two component response regulator | Complete |
| NEIS1549 | nitrite reductase, major outer membrane copper-containing protein | Complete |
| NEIS1573 | hypothetical protein | Incomplete |
| NEIS1574 | DNA transport competence protein | Incomplete |
| NEIS1615 | hypothetical protein | Complete |
| NEIS1621 | hypothetical protein | Incomplete |
| NEIS1623 | hypothetical protein | Incomplete |
| NEIS1630 | hypothetical protein | Complete |
| NEIS1672 | hypothetical protein | Incomplete |
| NEIS1727 | acetate kinase | Complete |
| NEIS1746 | hypothetical protein | Complete |
| NEIS1789 | MafA adhesin | Complete |
| NEIS1794 | putative mafS3 cassette | Incomplete |
| NEIS1800 | alternative toxic C-terminal extremity | Complete |
| NEIS1874 | phospho-2-dehydro-3-deoxyheptonate aldolase | Complete |
| NEIS1875 | hypothetical protein | Incomplete |
| NEIS1880 | DNA transport competence protein | Incomplete |
| NEIS1885 | thiol:disulfide interchange protein DsbA2; oxidoreductase | Incomplete |
| NEIS1901 | lacto-N-neotetraose biosynthesis glycosyl transferase | Incomplete |
| NEIS1939 | transcriptional regulator | Incomplete |
| NEIS1996 | DNA transport competence protein | Incomplete |
| NEIS2005 | putative permease | Incomplete |
| NEIS2043 | ThiF protein | Complete |
| NEIS2068 | hypothetical protein | Incomplete |

Supplemental Table 3: Diatabs reaction’s interpretation guide

| Diatabs | Positive | Negative |
| --- | --- | --- |
| Glucose | Yellow | Red/Orange-red |
| Maltose | Yellow | Red/Orange-red |
| Sucrose | Yellow | Red/Orange-red |
| Lactose | Yellow | Red/Orange-red |
| Gamma-Glutamyl Aminopeptidase (GGA)* | Red/Orange | Yellow |
| Leucine Aminopeptidase (LA)* | Orange/light orange | Yellow |
| proline Aminopeptidase (PA) * | Red/Orange | Yellow |
| Beta-Galactosidase (ONPG) | Yellow | Colourless |
| Tributyrin | Yellow/Yellow-orange | Red |

* Read after 4 hours. All the other tests were read after an overnight incubation

Supplemental Table 4: API-NH reaction’s interpretation guide

| Tests | Reactions | Positive | Negative |
| --- | --- | --- | --- |
| PEN | Penicillinase | Yellow/ Yellow-Green/ Yellow-Blue | Blue |
| GLU | Glucose Acidification | Yellow/Orange | Red/Red-Orange |
| FRU | Fructose Acidification | Yellow/Orange | Red/Red-Orange |
| MAL | Maltose Acidification | Yellow/Orange | Red/Red-Orange |
| SAC | Saccharose Acidification | Yellow/Orange | Red/Red-Orange |
| ODC | Ornithine Decarboxylase | Blue | Yellow-Green/Grey-Green |
| URE | Urease | Pink-Purple | Yellow |
| LIP | Lipase | Blue* | Colourless/ Pale Grey |
| PAL | Alkaline Phosphatase | Yellow | Colourless/ Pale Yellow |
| βGAL | β-Galactosidase | Yellow | Colourless |
| ProA | Proline Arylamidase** | Orange | Yellow/ Pale Orange |
| GGT | Gamma-Glutamyl Transferase | Orange | Yellow/ Pale Orange*** |
| IND | Indole | Pink | Colourless |

* With precipitate

** ProA is always negative when LIP was positive,

*** Yellow-Orange if PAL was positive
